# Epigenetic Scanning of *KEAP1* CpG Sites Uncovers New Molecular-Driven Patterns in Lung Adeno and Squamous Cell Carcinomas

**DOI:** 10.3390/antiox9090904

**Published:** 2020-09-22

**Authors:** Federico Pio Fabrizio, Tommaso Mazza, Stefano Castellana, Angelo Sparaneo, Lucia Anna Muscarella

**Affiliations:** 1 Laboratory of Oncology, Fondazione IRCCS Casa Sollievo della Sofferenza, San Giovanni Rotondo, 71013 Foggia, Italy; a.sparaneo@operapadrepio.it; 2 Unit of Bioinformatics, Fondazione IRCCS Casa Sollievo della Sofferenza, San Giovanni Rotondo, 71013 Foggia, Italy; t.mazza@operapadrepio.it (T.M.); s.castellana@operapadrepio.it (S.C.)

**Keywords:** KEAP1 (Kelch-like ECH-associated protein 1), methylation, NSCLC (non-small-cell lung cancer), prognostic marker

## Abstract

Background: The KEAP1/NRF2 (Kelch-like ECH-associated protein 1/nuclear factor erythroid 2–related factor 2) pathway modulates detoxification processes and participates in the resistance of solid tumors to therapy. Scientific evidence about the presence of genetic and epigenetic abnormalities of the *KEAP1* gene was firstly reported in non-small-cell lung cancer (NSCLC) and then described in other tumors. At present, the prognostic role of aberrant methylation at cytosine-guanine dinucleotide (CpG) sites of the *KEAP1* gene promoter is debated in NSCLC, and its correlation with transcriptional changes and protein levels remains to be defined in large sample cohorts. Methods: We evaluated and compared multiple KEAP1 omics data (methylation, transcript, and protein expression levels) from The Cancer Genome Atlas (TCGA) to explore the role of CpGs located in different portions of *KEAP1* and the correlation between methylation, transcription, and protein levels. Data from two subsets of lung adenocarcinoma (LUAD, *n* = 617) and lung squamous cell carcinoma (LUSC, *n* = 571) cohorts of NSCLC patients with different disease stages were evaluated. Results: We found that the methylation levels of many *KEAP1* CpGs at various promoter and intragenic locations showed a significant inverse correlation with the transcript levels. Interestingly, these results were limited to the *KRAS* wild-type LUSC and LUAD cohorts, whereas in LUAD the effect of the epigenetic silencing of *KEAP1* on its transcription was also observed in the *EGFR* mutated subpopulation. Conclusions: These results support the idea that the prognostic role of *KEAP1* CpG sites warrants more in-depth investigation and that the impact of their changes in methylation levels may differ among specific NSCLC histologies and molecular backgrounds. Moreover, the observed impact of epigenetic silencing on *KEAP1* expression in specific *KRAS* and *EGFR* settings may suggest a potential role of *KEAP1* methylation as a predictive marker for NSCLC patients for whom anti-EGFR treatments are considered.

## 1. Introduction

Lung cancer is the leading cause of cancer-related deaths worldwide. Due to the lack of symptoms in early-stage of the disease, most patients are diagnosed when the lung tumor is at an advanced stage, thus resulting in poor outcomes [1]. Hundreds of studies have been published on discovering new prognostic molecular factors beyond the tumor-node-metastasis (TNM) non-small-cell lung cancer (NSCLC) staging system, aiming to provide some new biological and clinical insights [2]. Interestingly, a significant translational impact in terms of increased risk of cancer progression and shorter overall survival was documented for alterations in the Kelch-like ECH-associated protein 1 (*KEAP1*) gene [3,4,5]. KEAP1 is a cytoplasmic anchor protein of Nuclear factor erythoid-2-related factor 2 (NRF2), encoded by the *NFE2L2* gene, which predominantly acts as a key regulator of antioxidant stress responses also in the context of tumor resistance and progression [6,7,8]. In cancer, loss of KEAP1 function leads to enhanced activity of NRF2 and antioxidant-related element (ARE)-driven gene expression, thus promoting cellular resistance to oxidative stress, rapid proliferation, and metabolic deregulation [9]. Among the genetic lesions that affect KEAP1/NRF2 activity, point mutations were the first reported mechanism of deregulation in NSCLC and other solid tumors [6,10]. Generally, they commonly affect the exonic regions of the *KEAP1* gene that code for the interaction sites between the Kelch/double-glycine repeat (DGR) domain of KEAP1 and the Nrf2-ECH homology (Neh2) domain of NRF2. Mutations in the *KEAP1* or *NFE2L2* genes are mutually exclusive and occur in NSCLC patients with a variable incidence (3.5–15% for *KEAP1*; 12–17% for *NFE2L2*), with the first ones mainly clustered with the lung adenocarcinoma (LUAD) histology [11,12,13]. In addition to the genetic lesions, epigenetic abnormalities, such as aberrant cytosine-guanine dinucleotide (CpG) methylation at the *KEAP1* promoter island, have been widely reported as one of the main mechanisms of *KEAP1* silencing in tumors [6]. Aberrant methylation at the *KEAP1* promoter was firstly described in human NSCLC cell lines and tissues and involves the CpGs grouped into one main island located near the transcriptional start site (TSS) [14]. In consequence, the observed effect of *KEAP1* methylation was to suppress gene expression by abrogating the Sp1 transcription factor binding sites in the promoter region [15]. This mechanism of *KEAP1* epigenetic silencing was also reported in neoplastic tissues of patients affected by NSCLC and carcinoid tumors, and it was associated with increased risk of lung cancer progression in surgically resected NSCLC patients [16,17]. In clear-cell renal cell carcinoma (ccRCC), the epigenetic modulation of *KEAP1* was shown to be the leading mechanism of KEAP1 deregulation, and it was able to strongly predict patient survival [18]. In primary breast cancers and pre-invasive lesions, aberrant *KEAP1* promoter methylation was seen to be associated to the estrogen receptor (ER)-positive status and was hypothesized to be a prognostic marker of mortality risk [19]. Aberrant *KEAP1* methylation was also reported in colorectal cancer cells and cancer tissues and was linked to a downregulation of its transcriptional activity and an upregulation of NRF2 and its target genes’ expression [20,21]. In pancreatic cancer cell lines, the suppression of KEAP1 protein by promoter methylation was demonstrated to be correlated with Ubiquitin-like containing PHD and RING finger domains 1 (UHRF1) increased expression, a scaffold protein for DNA methyltransferase 1 (DNMT1). Finally, in glioma *KEAP1,* methylation was inversely correlated with its transcription levels and reported as a predictor of patient outcome [22,23].

Despite the well-documented clinical impact of *KEAP1* and *NFE2L2* mutations [12], the role of aberrant *KEAP1* methylation was not fully elucidated in NSCLC, and its clinical prognostic significance in many solid tumors remains controversial [17]. Studies on chemoresistance suggested that selective inhibition of *KEAP1* methylation in adenocarcinoma cells could represent a marker of radiosensitizing effects in lung cancer [24]. More recently, our group observed that epigenetic deregulation of the KEAP1/NRF2 system by methylation at the *KEAP1* promoter could involve only some CpGs at the P1a region, thus suggesting that an evaluation of each single CpG methylation status might give more information and have more translational utility than a semi-quantitative evaluation of an entire CpG island [17,25]. Less information is available in NSCLC about additional mechanisms of epigenetic modulation, such as alteration in poorly investigated intragenic methylation sites of the *KEAP1* gene. The latest emerging roles of non-promoter CpG islands of cancer-related genes and the translational impact of KEAP1/NRF2 point mutations in predicting resistance to treatment further beg the question of the translational utility of *KEAP1* methylation in the clinical context. It is thus important to clarify if *KEAP1* methylation status at specific CpGs should be included or not in the molecular prognostic algorithm to optimize patient management and improve outcomes in terms of response to therapy and survival. 

To help address this question, we performed an integrated multi-omics data evaluation of *KEAP1* annotated CpGs using the available The Cancer Genome Atlas (TCGA) data from NSCLC datasets. More specifically, we investigated whether *KEAP1* CpG methylation sites correlated with *KEAP1* gene expression and KEAP1 protein levels in LUAD and lung squamous carcinoma (LUSC) histologies at different stages of the disease. 

## 2. Materials and Methods 

### 2.1. KEAP1 Promoter and Intragenic CpGs

We investigated a total of 16 CpGs of the *KEAP1* gene located inside or outside the well-known gene promoter region. All methylation data of CpGs are from Illumina 450K TGCA datasets, and their genomic positions are reported in Table 1. The respective fully converted DNA sequence of each *KEAP1* CpG island or single CpG site is available in Supplementary Materials (Supplemental Table S1).

**Figure 1:**
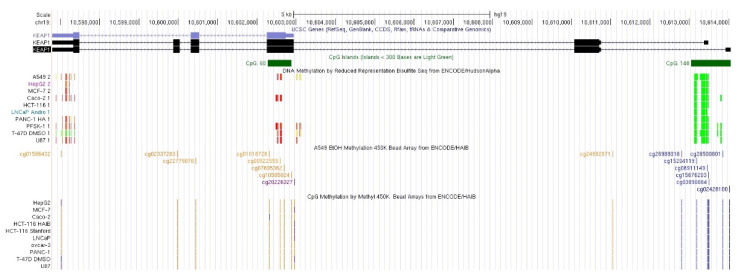
Schematic representation of the *KEAP1* gene structure within the human hg19 genome sequence. The first reference transcript (NM_203500) is located at chr19:10596796-10614054 (strand -, 17259 bp long), while the second one (NM_012289) is located at chr19:10596796-10613481 (16686 bp long). They encode for a 624 aa protein (NCBIID: NP_987096, Uniprot ID: Q14145). Annotation and methylation data retrieved by the University of California, Santa Cruz (UCSC) Genome Browser. From top to bottom: NCBI RefSeq and Consensus CDS tracks for *KEAP1* exon/intron structure; predicted CpG islands (“Regulation” >> “CpG Island” track); detected methylation sites (“Regulation” >> “ENCODE DNA Methylation tracks” >> “DNA Methylation by Reduced Representation Bisulfite Seq from ENCODE/Hudson Alpha” and “CpG Methylation by Methyl 450K Bead Arrays from ENCODE/HAIB”. Methylation status of genomic sites is represented through a color gradient vertical bars. Red-orange bars indicate strong methylation signals (i.e., a high relative number of methylated molecules vs. unmethylated molecules in bisulfite sequencing experiments); orange and blue bars indicate strong methylated vs. unmethylated status in 450K array experiments.

Many investigated CpGs are located in two main *KEAP1* CpG mapped islands. The first one (CpG island 1, CGI-1) comprises a long CpG-rich island of ~1.2 kb (chr19:10613047-10614280) that spans from the gene promoter region to intron 1, within the human hg19/GRCh37 genome sequence. The CGI-1 island includes a total of 148 CpGs, distributed in the P1 Region (-291-89) near the *KEAP1* TSS, and the P2 Region (-88+337), [25]. Four out of the 16 investigated CpGs fall in a shorter CpG island (CGI-2) that starts from chr19:10602253 and ends at 10602938, according to the University of California, Santa Cruz (UCSC) CpG island track (hg19/GRCh37), and includes a total of 60 CpGs (Figure 1).

### 2.2. TCGA Data Analysis

The TCGA *KEAP1* methylation and expression data of LUSC and LUAD datasets were directly pulled down from UCSC Xena public data hubs (https://xenabrowser.net). Clinical data were complemented and completed with information available from the GDC (Genomic Data Commons) Data Portal (https://portal.gdc.cancer.gov). These data include 617 LUAD patients (all stages) and 571 LUSC (all stages) patients. We also extrapolated the two subsets of LUAD (*n* = 128) and LUSC (*n* = 141) patients with non-metastatic disease from the whole cohorts (Table 2 and Table 3). Data related to *EGFR, KRAS*, and smoking status were also obtained, and patients were divided into the following cohorts: LUAD smokers (all stages, *n* = 508; early stages, *n* = 124), LUAD non-smokers (all stages, *n* = 14; early stages, *n* = 2); LUAD *EGFR* mutated (all stages, *n* = 63; early stages, *n* = 5), LUAD *EGFR* wild-type (all stages, *n* = 459; early stages, *n* = 98); LUAD *KRAS* mutated (all stages, *n* = 447; early stages, *n* = 101), LUAD *KRAS* wild-type (all stages, *n* = 75; early stages, *n* = 25). A total of 60 LUAD patients of all stages (95% *EGFR* mutated and 80% *KRAS* wild-type) shared the *EGFR* mutated/*KRAS* wild-type molecular subsets. Ninety-five LUAD subjects were not annotated with any information about *KRAS/EGFR* mutations and smoking habits. Finally, there were 15 LUSC *KRAS* mutated patients (all stages) and 246 LUSC *KRAS* wild-type patients (all stages). A set of 30 normal lung tissues was also included in our study to assess differences in CpG methylation levels. 

Specifically, the DNA methylation data were generated by Infinium Human Methylation 450K BeadChip microarrays and are stored in the Pan-Cancer Atlas Hub; gene expression data were obtained by RNA-Seq experiments and are available from the UCSC Toil RNAseq Recompute Compendium. Methylation data were available as beta-values, while expression data were available as TPM (Transcripts Per kilobase Million)-normalized reads counts. RPPA (Reverse phase protein array)-based protein expression data were retrieved from LinkedOmics (http://www.linkedomics.org).

### 2.3. Statistical Analysis

Clinical and histological characteristics of patients were reported as mean ± standard deviation (SD) or absolute frequencies and percentages for continuous and categorical variables, respectively. 

Correlation between *KEAP1* mRNA expression and all individual beta-values of KEAP1 in the TCGA datasets was assessed using Pearson’s correlation coefficient. Similarly, an overall assessment of correlation was calculated by aggregating the beta-values of all CpGs (average). Differential methylation was assessed using the Wilcoxon rank-sum test with continuity correction.

Results were deemed statistically significant when *p* was <0.05.

## 3. Results

### 3.1. KEAP1 Is Hypermethylated in Tumor Compared to Non-Neoplastic Tissues

For the analysis of *KEAP1* CpGs methylation within the TCGA cohort, Illumina Infinium Human Methylation 450 BeadChip beads (cg25801292, cg02428100, cg06911149, cg26500801, cg03890664, cg15676203, cg15204119, cg26988016, cg20226327, cg10505024, cg07695362, cg00522555, cg01018726, cg22779878, cg02337283, cg01586432) were used. 

**Figure 2:**
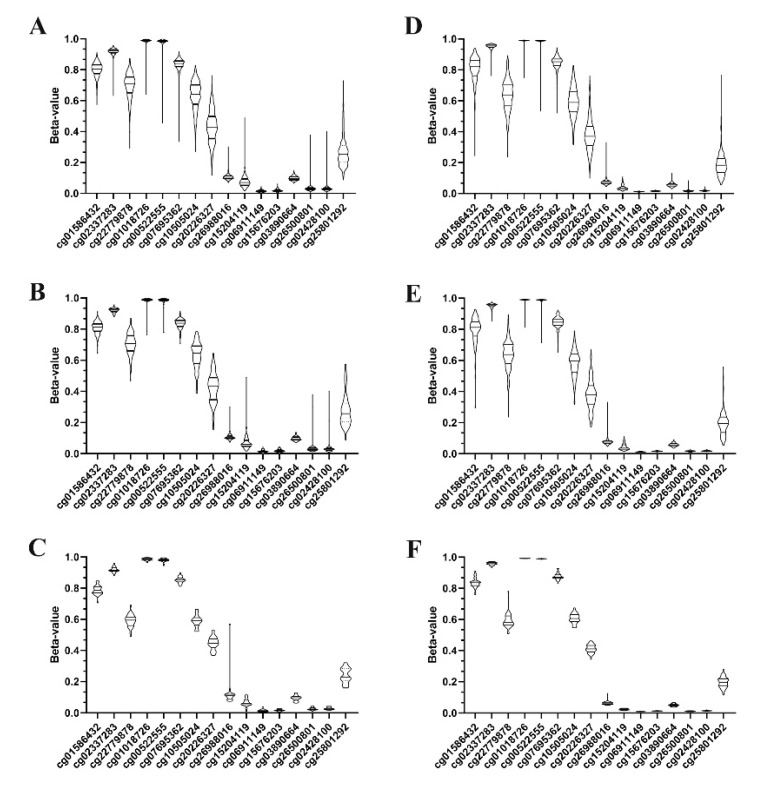
*KEAP1* per-CpG violin plots of beta-values of the all-stages (**A**: LUAD, **D**: LUSC), non-metastatic disease (**B**: LUAD, **E**: LUSC) and control cohorts (**C**: LUAD, **F**: LUSC). Violins report the median beta-values and interquartile ranges.

Both in non-neoplastic and in tumor tissues of LUAD and LUSC, CpG-sites targeted by beads located peripherally in the CpG-dense area of *KEAP1* (which includes the GCI-1 island) showed lower methylation levels, than the beads located in the central position of *KEAP1* gene, including the CGI-2 island; the only exception was cg25801292 (Figure 2). 

**Figure 3:**
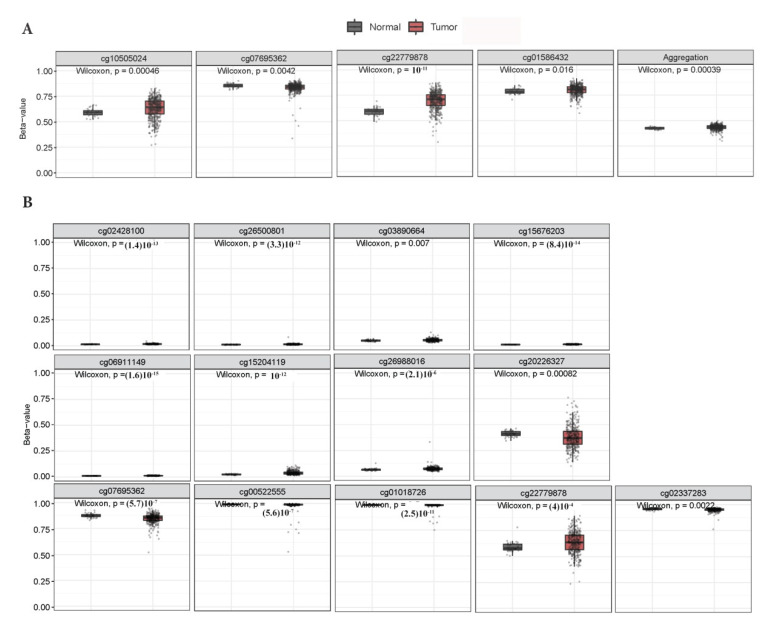
Box plots of differentially methylated *KEAP1* CpGs between all-stages cohort (**A**: LUAD, **B**: LUSC) and control.

Interestingly, *KEAP1* hypermethylation was found in tumors compared to non-neoplastic tissues of the LUAD all-stages cohort in 4/16 CpGs vs. 13/16 CpGs in the LUSC all-stages cohort (Figure 3). Statistically significant differences between tumor and non-neoplastic lung tissues were observed also at CpGs having low methylation levels and located at the CGI-1 island.

### 3.2. KEAP1 CpGs Methylation Inversely Correlates with mRNA Expression of KEAP1

**Figure 4:**
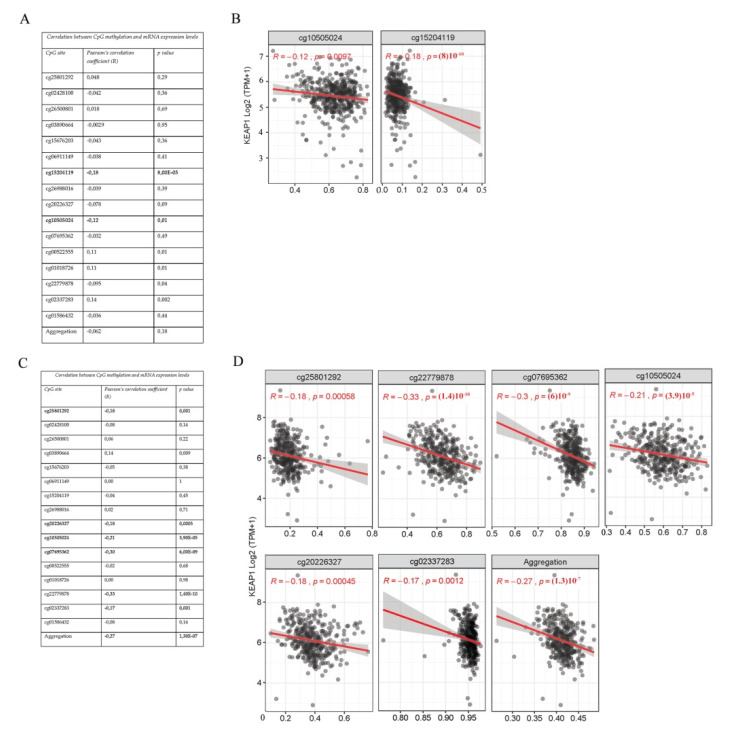
Correlation analysis between *KEAP1* methylation (450 K BeadChip array, beta-values) and its expression levels (RNA-Seq, TPM-normalized read counts) values from TCGA-LUAD all-stages (**A**) and LUSC all-stages (**C**) datasets. Scatter plots between β-values (x-axis) and expression values of *KEAP1* in LUSC (**B**) and LUAD (**D**). TCGA cohort includes the most significant CpG sites and aggregated data of all CpGs. Pearson’s correlation coefficient (*R*) and significance level (*p*) are reported for each plot.

Considering all-stages of LUAD patients, we found that only two predicted *KEAP1* CpG sites, are located in the CGI-1 and CGI-2 islands (cg15204119, *R* = −0,18, *p* = 8 × 10^−0.5^; cg10505024, *R* = −0,12, *p* = 0.0097), were significantly and inversely correlated with *KEAP1* mRNA levels (Figure 4A,B). More surprisingly, we observed a clear and inverse association between six *KEAP1* CpG sites (cg25801292, *R* = −0.18, *p* = 0.001; cg20226327, *R* = −0.18, *p* = 0.0005; cg10505024, *R* =−0.21, *p* =3.9 × 10^−0.5^; cg07695362, *R* = −0.3, *p* = 6 × 10^−0.9^; cg22779878, *R* = −0.33 *p* = 1.40 × 10^−10^; cg02337283, *R* = −0.17, *p* = 0.001) and mRNA levels in the LUSC group (all stages) (Figure 4C,D). As for the LUAD cohort, three out of these CpGs are located in the CGI-1 (cg25801292) and CGI-2 (cg10505024 and cg07695362) regions.

**Figure 5:**
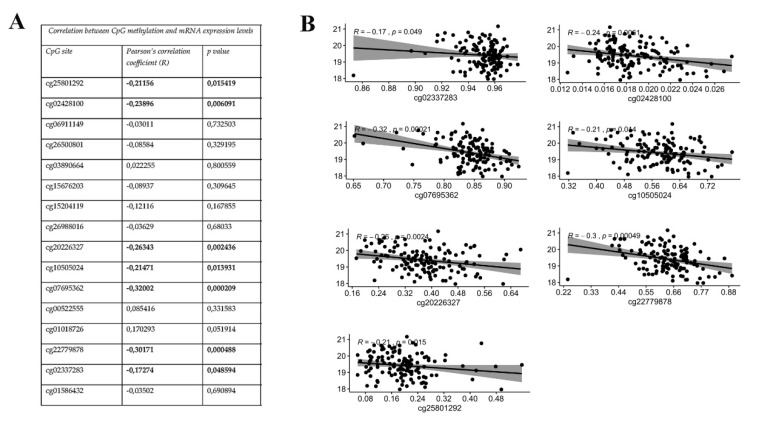
(**A**) Results from correlation analysis between *KEAP1* methylation (450 K BeadChip array, Beta-values) and its expression levels (RNA-Seq, TPM-normalized read counts). (**B**) Scatter plots between β-values (x-axis) and expression values of *KEAP1* in the TCGA LUSC non-metastatic disease dataset include the most significantly correlated CpG sites. Pearson’s correlation coefficient (*R*) and significance level (*p*) are also reported for each plot.

We also explored whether these same CpGs correlated with mRNA levels in LUAD and LUSC, but strictly related to non-metastastic disease. Almost half of the *KEAP1* CpG sites related to the LUSC cohort exhibited a significant correlation with KEAP1 expression (cg25801292 and cg02428100, exon 1-CGI-1; cg20226327, intron 2; cg10505024 and cg07695362, exon 3-CGI-2; cg22779878, exon 4; cg02337283, exon 5; Figure 5A,B),whereas *KEAP1* methylated CpG sites did not show any significant correlation in the LUAD non-metastatic cohort. Results were slightly different when the LUAD smoker all-stages cohort was examined, where a significant inverse correlation between *KEAP1* methylation and its expression emerged for the cg22779878 (exon 4), cg07695362 (exon 3-CGI-2), and cg15676203, (intron 1-CGI-1), (Supplemental Table S2). As a result of the small number of cases, no conclusive results were obtained for the LUAD non-smoker cohort. 

By contrast, a new, very intriguing scenario appeared when we considered the *EGFR* wild-type/mutated LUAD all-stages cohort. The effect of the *KEAP1* methylation on its transcript level was observed only in the *EGFR* mutated cohort, strictly related to the cg22779878 (exon 4), a block of 3 CpGs of the CGI-2 intragenic island (cg07695362, cg10505024, and cg20226327) an only one CpG of the CGI-1 promoter island (cg15676203). Conversely, in the subpopulation of *KRAS* wild-type all-stages LUAD, correlation was observed at the cg22779878 (exon 4), in only one CpG located in the CGI-2 intragenic island (cg07695362), but in three CpGs located in the CGI-1 promoter island (cg06911149, cg15676203, and cg03890664). The same pattern of clusterization of epigenetic events with *KRAS* wild-type status was confirmed when we examined the *KRAS* wild-type/mutated all-stages LUSC cohort (Supplemental Table S2). In this cohort, a large number of CpGs located in the CGI-1 (cg25801292, cg02428100, cg15204119, cg15676203) and CGI-2 islands (cg2022637, cg10505024, cg07695362, cg01018726) showed a negative correlation between *KEAP1* methylation and transcript expression, but only in the group of *KRAS* wild-type tumors (Supplemental Table S2).

**Figure 6:**
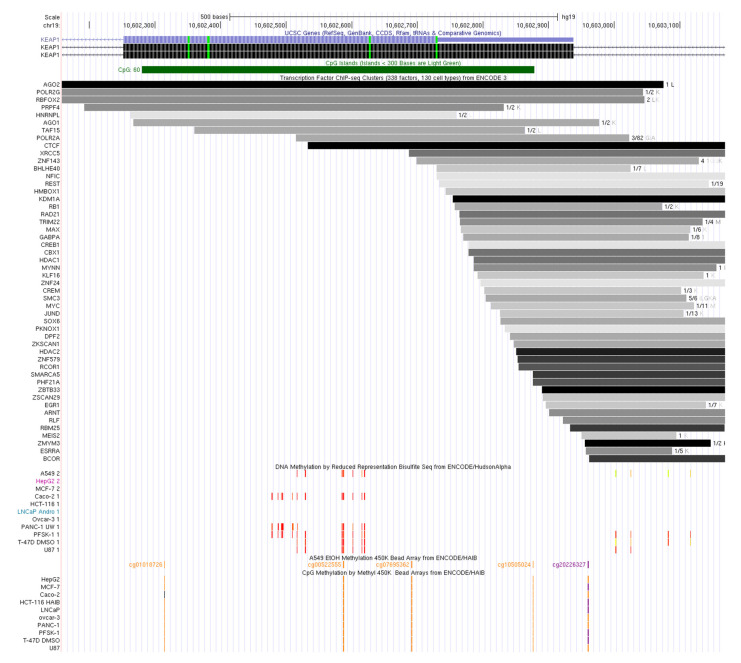
Schematic representation of *KEAP1* exon 3 (hg19 genome sequence). From top to bottom: NCBI RefSeq and Consensus CDS UCSC tracks for *KEAP1* exon/intron structure; TFBSs from the ENCODE Project; predicted CpG islands; detected methylation sites. The gray scale intensity of TF binding sites is proportional to the signal strength in the different tested cell lines (darker color indicates stronger signal). Red-orange bars indicate strong methylation signals; orange and blue bars indicate strong methylated vs. unmethylated status in 450K bead array experiments.

We then investigated the possibility that patterns of methylation may interfere with the transcription of *KEAP1* through modulation of the accessibility of transcription factor binding sites (TFBSs). Data of cell lines, derived from those tumors where methylation of *KEAP1* was described, were included into the computational analysis (Supplemental Table S3). Table 4 summarizes all the regulatory elements that co-localize with the CpG islands. CGI-1 was found to overlap with binding sites of E2F6 and CTCF (ENCODE Data release 3), and with functional elements of POL2RA, PHF8, MAX, and CTCF (ENCODE V2). Concerning the *KEAP1* exon 3 and the CGI-2, we observed the presence of CTCF sites at the position chr19: 1062751-10603103 (ENCODE V3 cell lines) and chr19:10602731-10603130 (ENCODE V2). Moreover, several cell line-specific TFBSs were located within this region (Figure 6), reinforcing the hypothesis that CGI-2 may influence the regulation of an alternative *KEAP1* transcript.

Finally, we investigated if *KEAP1* methylation at different CpG sites could be correlated with KEAP1 protein levels by using RPPA-based protein expression data were retrieved from LinkedOmics (http://www.linkedomics.org). No inverse correlation between CpGs methylation and KEAP1 protein levels was found in either the LUAD or the LUSC cohort (all-stage and non-metastatic disease stages, Supplemental Table S4).

**Figure 7:**
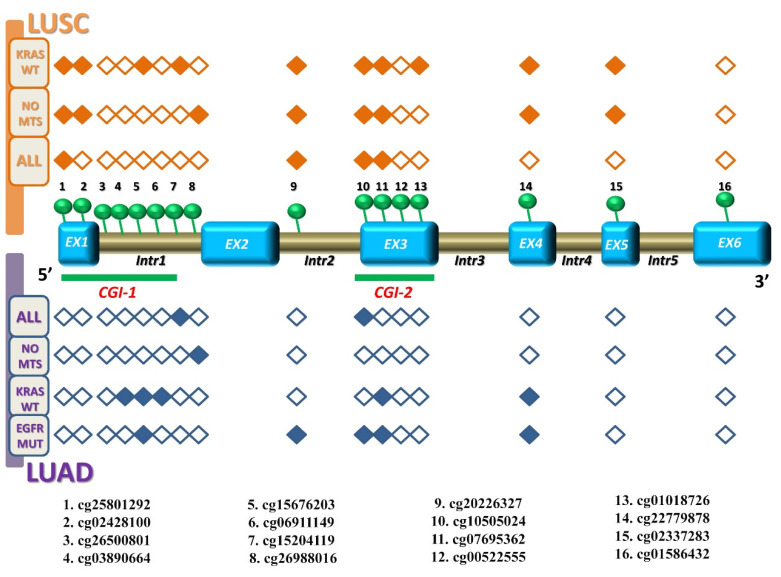
Correlation between *KEAP1* CpGs methylation and *KEAP1* transcript levels in LUSC and LUAD cohorts. The *KEAP1* gene structure is shown in the middle, including exons (sky blue boxes) and introns (brown boxes), as well as its two CpG islands: CGI-1 and CGI-2 (both underlined with green bars). A total of 16 CpG sites, marked with green circles, are indicated from 5′ to 3′ genomic localization (listed below). A filled rhombus, corresponding to a *KEAP1* CpG site, depicts a significant inverse correlation (*R* ≤ −0.1; *p* < 0.05) between methylation and transcription levels in LUSC (on the top, orange both for all, non-metastatic stages, and KRAS wild-type) and particularly in LUAD (on the bottom, blue both for all, non-metastatic stages, *KRAS* wild-type, and *EGFR* mutated), in contrast to an empty rhombus indicating no significant correlation. Abbreviations: EX, exon; Intr, intron; CGI-1, CpG Island 1; CGI-2, CpG Island 2; LUSC, lung squamous cell carcinoma; LUAD, lung adenocarcinoma; No-mts, non-metastatic disease stages.

Taken together, a novel interesting pattern of *KEAP1* CpG clusterization emerged by comparing the inverse correlation results between methylation and transcription levels in LUAD and LUSC at different stages (Figure 7). 

### 3.3. Identification of Gene Expression Signature Associated with KEAP1 Methylation in NSCLC

**Figure 8:**
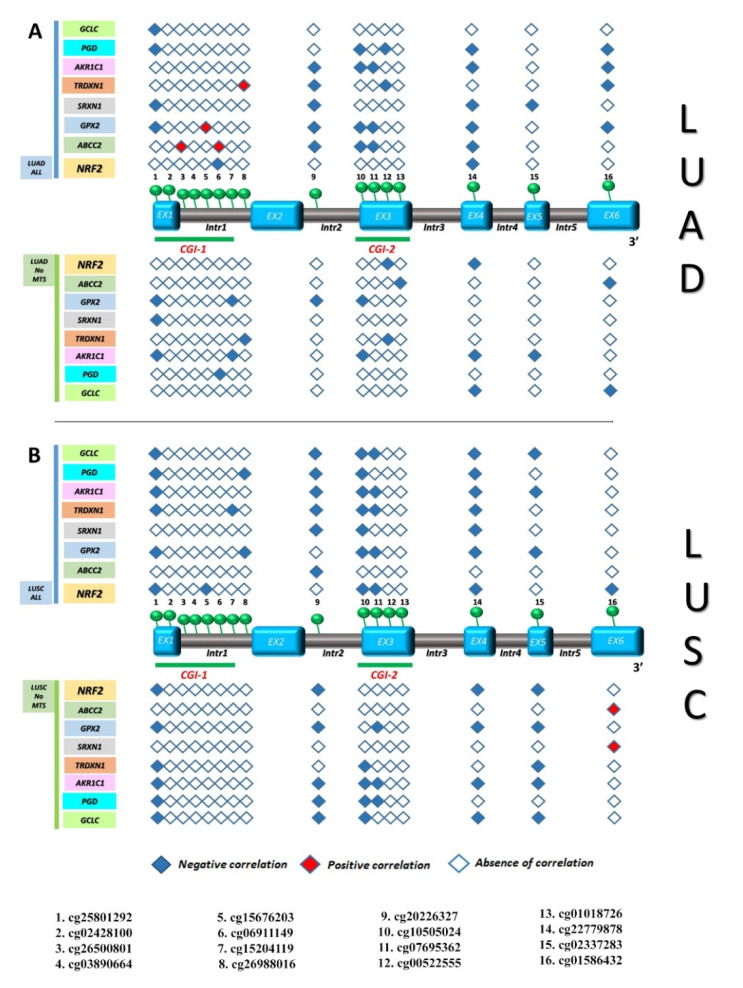
Correlation between *KEAP1* CpGs methylation and transcript levels of *NRF2* or its target genes in LUAD and LUSC cohorts. The *KEAP1* gene structure is shown in the middle, including exons (sky blue boxes) and introns (brown boxes), as well as its two CpG islands: CGI-1 and CGI-2 (both underlined with green bars). A total of 16 CpG sites, marked with green circles, are indicated from 5′ to 3′ genomic localization (listed below). A filled rhombus, corresponding to a *KEAP1* CpG site, depicts a significant inverse (blue square) or direct (red square) correlation (*R* ≤ −0.1; *p* < 0.05) between methylation and transcript levels of *NRF2* or its target genes in LUAD (**A**) and particularly in LUSC (**B**), in contrast to an empty rhombus indicating no significant correlation. Abbreviations: EX, exon; Intr, intron; CGI-1, CpG Island 1; CGI-2, CpG Island 2; LUSC, lung squamous cell carcinoma; LUAD, lung adenocarcinoma; No-mts, non-metastatic disease stages.

In order to investigate the effect of *KEAP1* methylation on NRF2 modulation, we correlated each *KEAP1* CpGs methylation level with the expression level of *NRF2* and some of its target genes that are involved in oxidative stress, oxidation-reduction processes, and the cellular response to oxidative stress: *GPX2*—*Glutathione Peroxidase 2, GCLC*—*Glutamate-Cysteine Ligase Catalytic Subunit, TXNRD1*—*Thioredoxin Reductase 1, AKR1C1*—*aldo-ketoreductase family 1, PGD*—*Phosphogluconate Dehydrogenase, SRXN1*—*Sulfiredoxin 1*, and *ABCC2—ATP Binding Cassette Subfamily C Member 2* [26,27]. An inverse, strong correlation between *KEAP1* methylation and mRNA expression levels of NRF2 and its targets was observed in both the LUAD and LUSC cohorts. In the LUAD cohort, a common pattern of correlation emerged between the methylation of cg227799878 (exon 4) and the expression of NRF2 and its targets. Many other CpGs, mainly located in the gene body regions, also showed a significant correlation with the expression levels of many NRF2 targets, such as *GPX2* and *AKR1C1* (Supplemental Table S5). In the LUSC cohort, a similar pattern of correlation was observed, but it was much more extended regarding the number of *KEAP1* CpGs that correlated with the expression of NRF2 and its target genes (Figure 8).

**Figure 9:**
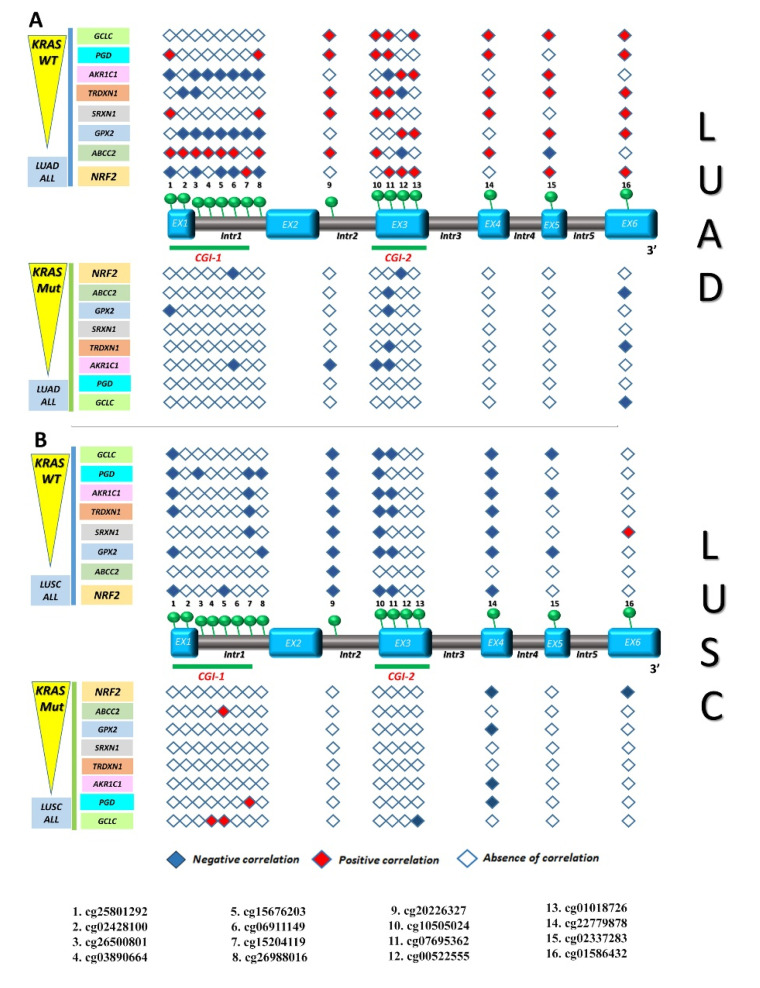
Correlation between *KEAP1* CpGs methylation and transcript levels of *NRF2* or its target genes in LUAD and LUSC KRAS wild-type/KRAS mutated subpopulations. The *KEAP1* gene structure, is shown in the middle, including exons (sky blue boxes) and introns (brown boxes), as well as two CpG islands: CGI-1 and CGI-2 (both underlined with green bars). A total of 16 CpG sites, marked with green circles, are indicated from 5′ to 3′ genomic localization (listed below). A filled rhombus, corresponding to a *KEAP1* CpG site, depicts a significant inverse (blue square) or direct (red square) correlation (*R* ≤ −0.1; *p* < 0.05) between methylation and transcript levels of *NRF2* or its target genes in LUAD *KRAS* wild-type/*KRAS* mutated (**A**) and particularly in LUSC *KRAS* wild-type/*KRAS* mutated (**B**), in contrast to an empty rhombus indicating no significant correlation. Abbreviations: EX, exon; Intr, intron; CGI-1, CpG Island 1; CGI-2, CpG Island 2; LUSC, lung squamous cell carcinoma; LUAD, lung adenocarcinoma; No-mts, non-metastatic disease stages.

Looking at the molecularly stratified NSCLC subpopulations, a strong positive correlation between *KEAP1* hypermethylation, mRNA expression levels of *NRF2,* and many ARE-driven target genes was observed in the LUAD *KRAS* wild-type subpopulation, both for many CpGs located in the CGI-1 island and in those located in the gene body. Among these, hypermethylation of cg15204119 (CGI-I island), cg07695362 (CGI-2 island), and cg22779878 (exon 4) correlated with both *KEAP1* mRNA downregulation and NRF2/ARE-driven target genes upregulation. This pattern of correlation was not found in the LUSC *KRAS* wild-type subpopulations, but an inverse correlation between *KEAP1* hypermethylation and mRNA expression levels of NRF2 and its ARE-driven target genes was observed for many CpGs (Figure 9 and Supplemental Table S6). Among these, hypermethylation of cg15204119, cg02428100, cg15676203, cg15204119 (all located in the CGI-I island), cg20226327 (intron 2), cg10505024 and cg07695362 (all located in the CGI-2 island), cg22779878 (exon 4), and cg02337283 (exon 5) correlated with both *KEAP1* mRNA downregulation and NRF2/ARE-driven target genes downregulation.

## 4. Discussion

As a result of their frequency and plasticity, epigenetic alterations are now emerging as innovative cancer biomarkers. Among these, DNA methylation is the most recognized one in lung tumors across neoplastic stages, since it has been observed that methylation patterns have undergone massive distortion in cancer cells, and it occurs early at different stages of the lung tumorigenesis process [28,29]. Similar to many genes that have CpG islands subjected to aberrant methylation, the *KEAP1* gene offers an interesting model to investigate epigenetic mechanisms in cancer cells, since the KEAP1/NRF2 pathway is directly linked to oxidative-stress and promotes chemo- and radio-resistance in different tumor types [6,30]. The presence of genetic and epigenetic abnormalities in this pathway, such as point mutations in functional domains of *KEAP1* and *NFE2L2* and methylation at the *KEAP1* promoter region, was firstly described in NSCLC and then widely reported in many solid tumors, with the hypothesis that the lack of *KEAP1* transcription induced high NRF2 activity and aberrant overexpression of ARE-driven target genes [6,10,16,18]. Actually, increasing attention to the *KEAP1* gene in lung cancer is mainly due to point mutations that show great translational impact in terms of increased risk of cancer progression, shorter overall survival and response of NSCLC patients’ to chemo and biological treatments [3,4,5,31]. This notion is also supported by a clear co-occurrence with *STK11* and *KRAS* gene alterations with a suggested synergic role of these genes in enhancement of tumorigenesis and lung cancer progression. Unlike *KEAP1* genetic lesions, the potential clinical utility of information related to *KEAP1* methylation has not been yet emerged. Methylation at the main CpG island located at the promoter gene region was widely investigated in the last decades, but without finding a real clinical implication in the context of lung cancer; this may be due to different reasons. Firstly, the published methylation analysis of *KEAP1* at promoter CpG island (named CGI-1 in the present work) has been frequently performed by semi-quantitative methods, such as real-time quantitative PCR. Even if rapid and sensitive, this method does not offer a detailed overview of each CpG function in the context of gene expression regulation. In support of this idea, our group recently observed that an evaluation of each CpG methylation status at the promoter region of *KEAP1* by pyrosequencing provides more information with a possible translational utility than the conventional semi-quantitative approach used until now [25]. Secondly, only some CpGs located at the promoter region and not all CpGs of *KEAP1* that could exert a strong regulatory effect on its transcription and protein levels have been yet investigated [26]. As for the intragenic CpGs island of many cancer-related genes, the function of CpGs located in the gene body of *KEAP1* has been poorly investigated. Near all current knowledge on transcriptional regulation by DNA methylation focuses on its role at the promoter of actively transcribed genes, since hypermethylation of the promoter clearly results in gene repression. Recently, accumulating scientific evidence has shown that changes in methylation patterns across a gene may exert a significant role in modulating the transcription of cancer-related genes [32]. In light of these observations, in this study we investigated all 16 *KEAP1* CpGs covered by 450K Illumina array, which are located in different regions of the gene. The data analyzed came from a large TCGA NSCLC cohort, and LUAD samples were analyzed separately from LUSC samples.

Our results confirmed that methylation events in NSCLC involve not only the CpGs at 5′ region of *KEAP1* gene, and showed, at first instance, that this event is apparently linked only to LUSC histology, with marginal effects in LUAD samples. These findings were corroborated by the fact that, regardless of non-metastatic and metastatic stages, no strong correlation appeared between *KEAP1* CpGs methylation and mRNA levels in LUAD, while this occurred instead in LUSC (both in all-stages and in non-metastatic stages). A novel and unexpected finding is that, when we stratified the LUAD population on the basis of the *EGFR* and *KRAS* status, the effects of *KEAP1* methylation on *KEAP1* transcription strongly re-emerged, strictly linked to an *EGFR* mutated and *KRAS* wild-type context. The link between the epigenetic silencing of *KEAP1* and *KRAS* wild-type status was also confirmed in LUSC, being present in the *KRAS* wild-type subpopulation and absent in *KRAS* mutated patients. 

Our results support the documented link between *KEAP1*, *EGFR,* and *KRAS* mutations and open the debate on the role of *KEAP1* methylation in the context of anti-EGFR treatments. For NSCLC patients whose tumors harbor mutations in *EGFR*, disruption of the KEAP1/NRF2 pathway is of the most recently reported mechanisms by which EGFR-tyrosine kinase inhibitors (EGFR-TKI) resistance occurs [5]. Coexisting mutations in the *KEAP1/NFE2L2/CUL3* genes were in fact reported to be associated with significantly decreased time to TKI treatment failure [4]. Taking into account our findings of a clear connection between *KEAP1* methylation and an *EGFR* mutated condition, we speculate that *KEAP1* methylation might represent an additional mechanism of TKI resistance in the context of oxidative stress modulation.

Apart from the mutual exclusivity of *EGFR* and *KRAS* mutations, the link between *KEAP1* methylation and a *KRAS* wild-type status deserves a specific consideration on the basis of two major scientific findings. First, oncogenic *KRAS* mutations were associated with chemoresistance and poor prognosis in NSCLC, also by controlling the *NFE2L2* gene transcription through a direct link of *KRAS* to a TPA response element (TRE) located in the *NFE2L2* promoter [33]. Second, *KRAS*-mutant tumors with coexisting *TP53* or *KEAP1* mutations were associated with a more aggressive tumor phenotype, and loss of *KEAP1* in tumors exhibits unique characteristics dictated by their cellular origin and metabolic program [34]. The finding that *KEAP1* transcript levels are modulated by methylation only in *KRAS* wild-type NSCLC might indicate that methylation is a *KRAS*-independent mechanism to modulate NRF2 levels in tumor cells. Alternatively, given that in our study no clear inverse correlation was observed between *KEAP1* methylation and *NFE2L2* transcription, the epigenetic silencing of *KEAP1* could represent an alternative or synergic way for NRF2 modulation of chemoresistance in NSCLC mediated by the *KRAS* gene status.

Methylation at different portions of the gene body appeared to have an active role in this context. Notably, even if the methylation pattern is different in tumor and non-neoplastic tissues, the methylation levels of the CpGs located in the 5′ portion of the gene are lower than those affecting the intragenic CpGs, thus suggesting that DNA methylation at these sites could have only a limited role in regulating tissue-specific transcription initiating from the canonical 5′ promoter region. In contrast, the high levels of methylation observed in all lung tumors at the intragenic CGI-2 and additional CpGs located in the gene body might reflect a functional role. In fact, we hypothesized that a non-canonical epigenetic regulation may exist for *KEAP1* at the exonic level, and specifically at exon 3, where a short CpG island (CGI-2 in our work) was predicted that starts from chr19:10602253 and ends at 10602938, according to the UCSC CpG Island track (human genome build: hg19). When hypermethylation occurs at this site, it might block the transcription machinery recruitment, thus producing two different transcripts. Specifically, the hypothetical model concerns the activation of *KEAP1* methylation at exon 3 that might impede the recruitment of CTCF, a multifunctional protein in genome regulation and gene expression, with consequent fast slipping of RNA Polymerase II [35]. At the protein level, an aberrant transcript without exon 3 should produce a shorter KEAP1 protein (624 amino acids in its canonical form), lacking amino acids 214-536. This would lead to partial loss of IVR and KELCH domains, both responsible for NRF2 interaction (fundamental arginine residues would be deleted, including Arg-380, Arg-415, and Arg-483). At the cellular level, intragenic *KEAP1* hypermethylation would increase the dosage of aberrant KEAP1 proteins at the expense of its canonical form, thereby contributing to enhance the levels of un-sequestered NRF2. To test this hypothesis, experimental assays are warranted to verify CpG methylation levels for the *KEAP1* genomic region, the presence of an exon 3-skipped transcript variant, and the dosage balance between normal and aberrant KEAP1 protein [36,37]. Surprisingly, we also observed that one *KEAP1* CpG (cg02337283), predicted in exon 5, was significantly recurrent in all bioinformatics analysis on non-metastatic LUAD and LUSC correlations; we thus wonder if DNA methylation might facilitate *KEAP1* exon inclusion in other settings by recruiting methyl-binding domain (MBD) proteins [38,39]. In contrast to the transcript level, no correlation was observed between methylation and protein levels. One explanation for this is that the proteome was probed using protein array Reverse Phase Protein Assay (RPPA) technology and that the antibodies-based analyses are inherently limited because of reduced coverage and inability to easily compare across proteins due to differential binding effects [40].

Strong correlations were observed between many CpGs located both in the *KEAP1* promoter and in the *KEAP1* gene body CGI and the mRNA transcript levels of *NRF2* and some of its target genes, both in LUAD and in LUSC; this evidence confirms the strong link between epigenetic *KEAP1* silencing and NRF2 activity modulation. Looking at different *EGFR/KRAS* subpopulations, a high level of methylation seems to be differently correlated in LUSC and LUAD with NRF2 mRNA levels and the transcription activity ARE-genes. Globally, a complex link emerges between *KEAP1* methylation and NRF2 deregulation that needs to be confirmed on large independent NSCLC cohorts.

## 5. Conclusions

Epigenetic deregulation has been increasingly recognized as one of the major mechanisms of the *KEAP1* gene deregulation in lung cancer. Our findings broaden the current knowledge on this topic and open the debate from multiple points of view. *KEAP1* methylation was described in lung many years ago and was mainly investigated at promoter region using a semi-quantitative approach, rather than a more detailed approach that might give more information with possible translational utility. This could represent one of the reasons why no strong clinical correlations have emerged in lung tumors, but this hypothesis warrants testing by high-throughput studies in large NSCLC cohorts. The intersection between the *KEAP1* methylation event and the *EGFR* mutant/*KRAS* wild-type status suggests a possible translational impact in terms of shorter overall survival and clinical benefit to chemotherapeutic and targeted biological treatments of patients with NSCLC. Therefore, it may be of interest to scan for this epigenetic event in specific oncogenic-addicted NSCLC patients, taking into consideration that the potential inclusion of *KEAP1* methylation in a molecular predictive/prognostic algorithm would first need a clinically validated cut-off setting specific for lung cancer.

*KEAP1* promoter methylation appears to have an unusual impact on NRF2 and its target genes’ expression, but no prior evidence about this has been published. Therefore, a conclusive demonstration of a potential non-canonical role of KEAP1 in modulating the NRF2 pathway warrants further investigation. Overall, it is hoped that functional validation of our results in cellular models and in independent lung cancer cohorts will contribute to rapidly translate these molecular results into the clinical context. 

## Figures and Tables

**Table 1:** List of investigated *KEAP1* exonic and intronic cytosine-guanine dinucleotide (CpG) sites.

Gene Symbol	CpG	Chromosome	Exonic/Intronic Location	Strand	Start	End
*KEAP1*	cg25801292	chr19	Exon 1	+	10614272	10614273
*KEAP1*	cg02428100	chr19	Exon 1	−	10614022	10614023
*KEAP1*	cg26500801	chr19	Intron 1	−	10613855	10613856
*KEAP1*	cg03890664	chr19	Intron 1	+	10613492	10613493
*KEAP1*	cg15676203	chr19	Intron 1	+	10613488	10613489
*KEAP1*	cg06911149	chr19	Intron 1	+	10613456	10613457
*KEAP1*	cg15204119	chr19	Intron 1	+	10613180	10613181
*KEAP1*	cg26988016	chr19	Intron 1	+	10612802	10612803
*KEAP1*	cg20226327	chr19	Intron 2	−	10602960	10602961
*KEAP1*	cg10505024	chr19	Exon 3	+	10602877	10602878
*KEAP1*	cg07695362	chr19	Exon 3	−	10602691	10602692
*KEAP1*	cg00522555	chr19	Exon 3	-	10602587	10602588
*KEAP1*	cg01018726	chr19	Exon 3	−	10602314	10602315
*KEAP1*	cg22779878	chr19	Exon 4	+	10600446	10600447
*KEAP1*	cg02337283	chr19	Exon 5	+	10599976	10599977
*KEAP1*	cg01586432	chr19	Exon 6	−	10597016	10597017

CpGs located at *KEAP1* CGI-1 (P1 and P2 regions) and CGI-2 3 islands are marked in bold. Chromosome 19p13.2 position is referred NM_203500.2, GRCh37/hg19 release. CpG Island Promoter position (including P1 region -291-89 and P2 region -88+337 from TSS): chr19:10613047-10614280 (Genomic Size: 1234; CpG count: 148). CpG Island exon 3 position: chr19:10602281-10602878 (Genomic Size: 598; CpG count: 60).

**Table 2:** Clinical-pathological data of lung adenocarcinoma (LUAD) from The Cancer Genome Atlas (TCGA) dataset.

Characteristics	Total	Characteristics	Total
All Stages		Non-Metastatic Disease	
Cohort size (*n*)	617	128	
Age at diagnosis Mean ± devst	65.94 ± 9.88	Age at diagnosis Mean ± devst	64.55 ± 9.82
Sex		Sex	
Male	284 (46%)	Male	60 (46.9%)
Female	333 (54%)	Female	68 (53.1%)
Race		Race	
White	465 (75.4%)	White	101 (78.9%)
Black or African American	59 (9.6%)	Black or African American	19 (14.8%)
Asian	10 (1.6%)	Asian	2 (1.6%)
NA	83 (13.5%)	NA	6 (4.7%)
Number of packs/year Mean ± SD	41.63 ± 27.22	Number of packs/year Mean ± SD	41.62 ± 24.71
Stage of disease		Stage of disease	
Stage I	5 (0.8%)	Stage I	1 (0.8%)
Stage IA	164 (26.6%)	Stage IA	47 (36.7%)
Stage IB	163 (26.4%)	Stage IB	30 (23.4%)
Stage II	1 (0.2%)	Stage II	1 (0.8%)
Stage IIA	63 (10.2%)	Stage IIA	22 (17.2%)
Stage IIB	87 (14.1%)	Stage IIB	14 (10.9%)
Stage IIIA	98 (15.9%)	Stage IIIA	13 (10.2%)
Stage IV	28 (4.5%)		
NA	8 (1.3%)		

SD, standard deviation.

**Table 3:** Clinical-pathological data of lung squamous cell carcinoma (LUSC) from the TCGA dataset.

Characteristics	Total	Characteristics	Total
All Stages		Non-Metastatic Disease	
Cohort size (*n*)	571	141	
Age at diagnosis Mean ± SD	67.65 ± 8.58	Age at diagnosis Mean ± SD	65.91 ± 8.66
Sex		Sex	
Male	424 (74.3%)	Male	112 (79.4%)
Female	147 (25.7%)	Female	29 (20.6%)
Race		Race	
White	402 (70.4%)	White	124 (87.9%)
Black or African American	40 (7%)	Black or African American	4 (2.8%)
Asian	10 (1.8%)	Asian	4 (2.8%)
NA	119 (20.8%)	NA	9 (6.4%)
Number of packs/year Mean ± SD	53.62 ± 32.12	Number of packs/year Mean ± SD	49.29 ± 25.24
Stage of disease		Stage of disease	
Stage I	5 (0.9%)	Stage IA	29 (20.6%)
Stage IA	107 (18.7%)	Stage IB	40 (28.4%)
Stage IB	172 (30.1%)	Stage IIA	30 (21.3%)
Stage II	3 (0.5%)	Stage IIB	24 (17%)
Stage IIA	75 (13.1%)	Stage IIIA	18 (12.8%)
Stage IIB	108 (18.9%)		
Stage III	3 (0.5%)		
Stage IIIA	66 (11.6%)		
Stage IIIB	21 (3.7%)		
Stage IV	7 (1.2%)		
NA	4 (0.7%)		

SD, standard deviation.

**Table 4:** Regulatory features co-localizing with *KEAP1* CpGs.

CpG					Regulatory Feature				
Exonic/Intronic Location	Strand	Start	End	CpG	Feat Start	Feat End	Name	Score	UCSC Track
Exon 1	+	10614272	10614273	cg25801292	10613910	10614318	E2F6	1000	A
Exon 1	+	10614272	10614273	cg25801292	10613919	10614347	E2F6_(H-50)	1000	A
Exon 1	−	10614022	10614023	cg02428100	10613910	10614318	E2F6	1000	A
Exon 1	−	10614022	10614023	cg02428100	10613919	10614347	E2F6_(H-50)	1000	A
Intron 2	−	10602960	10602961	cg20226327	10602751	10603103	CTCF	1000	A
Exon 3	+	10602877	10602878	cg10505024	10602751	10603103	CTCF	1000	A
Exon 1	+	10614272	10614273	cg25801292	10612580	10614352	POLR2A	1000	B
Exon 1	+	10614272	10614273	cg25801292	10612955	10614326	PHF8	1000	B
Exon 1	+	10614272	10614273	cg25801292	10613272	10614430	MAX	1000	B
Exon 1	+	10614272	10614273	cg25801292	10613814	10614366	E2F6	1000	B
Exon 1	-	10614022	10614023	cg02428100	10612580	10614352	POLR2A	1000	B
Exon 1	−	10614022	10614023	cg02428100	10612955	10614326	PHF8	1000	B
Exon 1	−	10614022	10614023	cg02428100	10613272	10614430	MAX	1000	B
Exon 1	−	10614022	10614023	cg02428100	10613814	10614366	E2F6	1000	B
Intron 2	−	10602960	10602961	cg20226327	10602731	10603130	CTCF	1000	B
Exon 3	+	10602877	10602878	cg10505024	10602731	10603130	CTCF	1000	B
Exon 3	−	10602691	10602692	cg07695362	10602731	10603130	CTCF	1000	B
Exon 5	+	10599976	10599977	cg02337283	10599967	10599986	V$GR_Q6	850	C

For each overlapping feature, the chromosomal start/end, the transcription factor name and scores are provided. Only high-confidence features (score > 800; maximum score = 1000) are reported. UCSC track A is related to transcription factor binding sites (TFBSs) from the ENCODE Project Release 3 (wgEncodeRegTfbsClusteredV3); track B is related to TFBS from the ENCODE Project Release 2 (wgEncodeRegTfbsClusteredV2); track C is related to a collection of computationally predicted TFBS (tfbsConsSites); conservation scores are computed through the Transfac Matrix and Factor Database v7.0, considering three species (human, mouse, rat). “V$GR_Q6” refers to a binding matrix associated with the NR3C1 receptor.

## Supplementary Materials

The following are available online at https://www.mdpi.com/2076-3921/9/9/904/s1, Table S1: List of methylation-converted sequences for each predicted *KEAP1* CpG site; Table S2: Results from correlation analysis between *KEAP1* methylation levels and its expression related to the *KRAS/EGFR* and smoking status. Table S3: Details on cancer cell lines used for in silico prediction analysis at exon3 CpG island (CGI-2). Table S4: Significant *KEAP1* CpG site in non-metastatic cohort. Table S5: Results from correlation analysis between *KEAP1* methylation levels and expression of NRF2 and ARE-targets. Table S6: Results from correlation analysis between *KEAP1* methylation levels and expression of NRF2 and ARE-targets in LUAD and LUSC EGFR/KRAS subpopulations.
