# Supplementary material for: Epigenetic Scanning of KEAP1 CpG Sites Uncovers New Molecular-Driven Patterns in Lung Adeno and Squamous Cell Carcinomas
Source: Antioxidants (Basel). 2020 Sep 22;9(9):904. doi: 10.3390/antiox9090904 (PMC7554999; doi:10.3390/antiox9090904)
Supplement: Supplementary file 1 [file antioxidants-09-00904-s001.pdf]

Table S1. List of methylation-converted sequences for each predicted *KEAP1* CpG site.

**CpG Island**	**sequence**
**cg25801292**	tggtccggcggc
**cg02428100**	cgggacgcccga
**cg26500801**	ccacccgcgcac
**cg03890664**	gcgcccgccgtg
**cg15676203**	ccgggcgcccgc
**cg06911149**	gagcgcgcgcag
**cg15204119**	gctcccgggcca
**cg26988016**	cactgcgcaggg
**cg20226327**	tgggacgggctg
**cg10505024**	cgtcccggctga
**cg07695362**	tgcagcgggagt
**cg00522555**	gcccgcggtgta
**cg01018726**	gccatcgatgac
**cg22779878**	ggagacgattga
**cg02337283**	ctccacgctgtt
**cg01586432**	gctgccggcatg

Supplemental Table S2.

Results from correlation analysis between KEAP1 methylation levels and its expression related to the *KRAS* status (**A** and **B**) in LUSC and LUAD (**C**, **D**, **E**, **F**).
Results from correlation analysis between KEAP1 methylation levels and its expression related to the *EGFR* status in LUAD (**G, H**, **I**, **L**).
Results from correlation analysis between KEAP1 methylation levels and its expression related to the smoking status in LUAD (**M, N, O, P**)

A_LUSC_All stages_*KRAS* mutated cohort

**CpG***	**CpG**	**P value**	**R**
1	cg25801292	0,96	0,01
2	cg02428100	0,69	0,11
3	cg26500801	0,09	0,45
4	cg03890664	0,11	0,43
5	cg15676203	0,36	0,25
6	cg06911149	0,14	0,40

7	cg15204119	0,14	0,40
8	cg26988016	0,05	0,53
9	cg20226327	0,81	-0,07
10	cg10505024	0,80	-0,07
11	cg07695362	0,04	-0,54
12	cg00522555	0,25	-0,32
13	cg01018726	0,15	-0,39
14	cg22779878	0,51	-0,19
15	cg02337283	0,34	-0,27
16	cg01586432	0,29	-0,29

**CpG number refers to the position of CpGs indicated in the legend of Figure 7.*

B_LUSC_All stages_*KRAS* wild-type cohort

CpG*	CpG	P value	R
1	cg25801292	0,00	-0,18
2	cg02428100	0,00	-0,18
3	cg26500801	0,38	-0,05
4	cg03890664	0,18	0,07
5	cg15676203	0,03	-0,12
6	cg06911149	0,07	-0,10
7	cg15204119	0,00	-0,18
8	cg26988016	0,92	0,01
9	cg20226327	0,00	-0,28
10	cg10505024	0,00	-0,21
11	cg07695362	0,00	-0,26
12	cg00522555	0,50	0,04
13	cg01018726	0,01	0,13
14	cg22779878	0,00	-0,35
15	cg02337283	0,03	-0,12
16	cg01586432	0,15	-0,08

**CpG number refers to the position of CpGs indicated in the legend of Figure 7.*

C_LUAD_All Stages_*KRAS* mutated cohort

CpG	CpG	P value	R
1	cg25801292	0,99	0,00
2	cg02428100	0,41	0,11
3	cg26500801	0,95	-0,01
4	cg03890664	0,18	0,17
5	cg15676203	0,93	-0,01
6	cg06911149	0,62	0,06
7	cg15204119	0,57	0,07
8	cg26988016	0,70	0,05
9	cg20226327	0,24	-0,15
10	cg10505024	0,39	-0,11
11	cg07695362	0,63	-0,06

12	cg00522555	0,55	-0,08
13	cg01018726	0,40	-0,11
14	cg22779878	0,12	-0,20
15	cg02337283	0,36	0,12
16	cg01586432	0,14	-0,19

**CpG number refers to the position of CpGs indicated in the legend of Figure 7.*

D_LUAD_All Stages_*KRAS* wild-type cohort

CpG	CpG	P value	R
1	cg25801292	0,45	-0,04
2	cg02428100	0,05	-0,10
3	cg26500801	0,61	-0,03
4	cg03890664	0,03	-0,11
5	cg15676203	0,01	-0,13
6	cg06911149	0,02	-0,12
7	cg15204119	0,47	-0,04
8	cg26988016	0,64	-0,02
9	cg20226327	0,08	-0,09
10	cg10505024	0,08	-0,09
11	cg07695362	0,02	-0,12
12	cg00522555	0,08	0,09
13	cg01018726	0,92	-0,01
14	cg22779878	0,01	-0,12
15	cg02337283	0,12	0,08
16	cg01586432	0,92	0,00

**CpG number refers to the position of CpGs indicated in the legend of Figure 7.*

E_LUAD_Non Metastatic_*KRAS* mutated cohort

CpG	CpG	P value	R
1	cg25801292	0,97	-0,01
2	cg02428100	0,57	0,14
3	cg26500801	0,77	-0,07
4	cg03890664	0,54	0,16
5	cg15676203	0,22	-0,30
6	cg06911149	0,93	0,02
7	cg15204119	0,77	0,08
8	cg26988016	0,52	-0,16
9	cg20226327	0,87	0,04
10	cg10505024	0,88	0,04
11	cg07695362	0,64	-0,12
12	cg00522555	0,72	0,09
13	cg01018726	0,70	0,10
14	cg22779878	0,93	-0,02
15	cg02337283	0,04	0,49
16	cg01586432	0,69	-0,10

**CpG number refers to the position of CpGs indicated in the legend of Figure 7.*

F_LUAD_Non Metastatic_*KRAS* wild-type cohort

CpG	CpG	P value	R
1	cg25801292	0,13	-0,15
2	cg02428100	0,47	-0,07
3	cg26500801	0,70	-0,04
4	cg03890664	0,05	-0,20
5	cg15676203	0,38	-0,09
6	cg06911149	0,52	-0,06
7	cg15204119	0,28	-0,11
8	cg26988016	0,05	-0,20
9	cg20226327	0,64	-0,05
10	cg10505024	0,90	-0,01
11	cg07695362	0,10	-0,16
12	cg00522555	0,06	0,18
13	cg01018726	0,55	0,06
14	cg22779878	0,70	-0,04
15	cg02337283	0,11	0,16
16	cg01586432	0,23	0,12

**CpG number refers to the position of CpGs indicated in the legend of Figure 7.*

G_LUAD_All stages_*EGFR* mutated cohort

CpG	CpG	P value	R
1	cg25801292	0,61	-0,03
2	cg02428100	0,12	-0,08
3	cg26500801	0,82	-0,01
4	cg03890664	0,11	-0,08
5	cg15676203	0,03	-0,11
6	cg06911149	0,09	-0,09
7	cg15204119	0,36	-0,05
8	cg26988016	0,75	-0,02
9	cg20226327	0,01	-0,13
10	cg10505024	0,01	-0,12
11	cg07695362	0,01	-0,13
12	cg00522555	0,20	0,07
13	cg01018726	0,82	-0,01
14	cg22779878	0,00	-0,15
15	cg02337283	0,15	0,07
16	cg01586432	0,93	0,00

**CpG number refers to the position of CpGs indicated in the legend of Figure 7.*

H_LUAD_All stages_*EGFR* wild-type cohort

CpG	CpG	P value	R
1	cg25801292	0,36	-0,12
2	cg02428100	0,35	-0,13
3	cg26500801	0,20	-0,17
4	cg03890664	0,94	-0,01
5	cg15676203	0,05	-0,26
6	cg06911149	0,09	-0,23
7	cg15204119	0,36	0,12
8	cg26988016	0,94	0,01
9	cg20226327	0,25	0,15
10	cg10505024	0,21	0,17
11	cg07695362	0,77	-0,04
12	cg00522555	0,24	0,16
13	cg01018726	0,79	0,04
14	cg22779878	0,85	-0,02
15	cg02337283	0,12	0,21
16	cg01586432	0,48	-0,09

**CpG number refers to the position of CpGs indicated in the legend of Figure 7.*

I_LUAD_ Non Metastatic_*EGFR* mutated cohort

of CpG	CpG	Pvalue	R
1	cg25801292	0,13	-0,14
2	cg02428100	0,80	-0,02
3	cg26500801	0,74	-0,03
4	cg03890664	0,05	-0,18
5	cg15676203	0,24	-0,11
6	cg06911149	0,59	-0,05
7	cg15204119	0,61	-0,05
8	cg26988016	0,05	-0,18
9	cg20226327	0,65	-0,04
10	cg10505024	0,96	-0,01
11	cg07695362	0,07	-0,17
12	cg00522555	0,06	0,17
13	cg01018726	0,45	0,07
14	cg22779878	0,71	-0,03
15	cg02337283	0,03	0,20
16	cg01586432	0,30	0,10

**CpG number refers to the position of CpGs indicated in the legend of Figure 7.*

L_LUAD_ Non Metastatic_*EGFR* wild-type cohort

CpG	CpG	P value	R
1	cg25801292	1,00	0,50
2	cg02428100	1,00	-0,50
3	cg26500801	1,00	-0,50
4	cg03890664	0,33	1,00
5	cg15676203	1,00	-0,50
6	cg06911149	1,00	-0,50
7	cg15204119	0,33	-1,00
8	cg26988016	1,00	-0,50
9	cg20226327	1,00	-0,50
10	cg10505024	1,00	-0,50
11	cg07695362	1,00	-0,50
12	cg00522555	1,00	0,00
13	cg01018726	1,00	0,50
14	cg22779878	0,33	-1,00
15	cg02337283	1,00	0,50
16	cg01586432	0,33	-1,00

**CpG number refers to the position of CpGs indicated in the legend of Figure 7.*

M_LUAD_All stages_smoking cohort

CpG	CpG	P value	R
1	cg25801292	0,309	-0,049
2	cg02428100	0,079	-0,084
3	cg26500801	0,582	-0,026
4	cg03890664	0,151	-0,069
5	cg15676203	0,005	-0,133
6	cg06911149	0,042	-0,097
7	cg15204119	0,414	-0,039
8	cg26988016	0,679	-0,020
9	cg20226327	0,062	-0,089
10	cg10505024	0,059	-0,090
11	cg07695362	0,012	-0,120
12	cg00522555	0,094	0,080
13	cg01018726	0,933	-0,004
14	cg22779878	0,004	-0,136
15	cg02337283	0,053	0,093
16	cg01586432	0,733	-0,016

**CpG number refers to the position of CpGs indicated in the legend of Figure 7.*

N_LUAD_All stages_non-smoking cohort

CpG	CpG	P value	R
1	cg25801292	0,18	0,38
2	cg02428100	1,00	0,00
3	cg26500801	0,50	0,20
4	cg03890664	0,16	-0,40
5	cg15676203	0,49	0,20
6	cg06911149	0,94	-0,02
7	cg15204119	0,30	0,30
8	cg26988016	0,51	-0,19
9	cg20226327	0,50	-0,20
10	cg10505024	0,36	-0,27
11	cg07695362	0,53	-0,18
12	cg00522555	0,30	0,30
13	cg01018726	0,28	0,31
14	cg22779878	1,00	0,00
15	cg02337283	0,35	-0,27
16	cg01586432	0,83	0,06

**CpG number refers to the position of CpGs indicated in the legend of Figure 7.*

O_LUAD_Non metastatic_smoking cohort

CpG	CpG	P value	R
1	cg25801292	0,11	-0,15
2	cg02428100	0,48	-0,07
3	cg26500801	0,62	-0,05
4	cg03890664	0,11	-0,15
5	cg15676203	0,19	-0,12
6	cg06911149	0,44	-0,07
7	cg15204119	0,46	-0,07
8	cg26988016	0,03	-0,20
9	cg20226327	0,56	-0,05
10	cg10505024	0,81	-0,02
11	cg07695362	0,09	-0,15
12	cg00522555	0,05	0,18
13	cg01018726	0,42	0,07
14	cg22779878	0,55	-0,05
15	cg02337283	0,04	0,19
16	cg01586432	0,33	0,09

**CpG number refers to the position of CpGs indicated in the legend of Figure 7.*

P_LUAD_Non metastatic_non-smoking cohort

CpG	CpG	P value	R
1	cg25801292	1,0	1,0
2	cg02428100	1,0	1,0
3	cg26500801	1,0	1,0
4	cg03890664	1,0	-1,0
5	cg15676203	1,0	-1,0
6	cg06911149	1,0	1,0
7	cg15204119	1,0	-1,0
8	cg26988016	1,0	1,0
9	cg20226327	1,0	1,0
10	cg10505024	1,0	1,0
11	cg07695362	1,0	-1,0
12	cg00522555	1,0	1,0
13	cg01018726	1,0	1,0
14	cg22779878	1,0	1,0
15	cg02337283	1,0	1,0
16	cg01586432	1,0	-1,0

**CpG number refers to the position of CpGs indicated in the legend of Figure 7.*

Supplemental Table 3. Details on cancer cell lines used for in silico prediction analysis at exon3 CpG island (CGI-2).

Cell line	Derivation	Tissue	Details

A459	lung carcinoma tissue	epithelium	https://genome.ucsc.edu/cgi-bin/hgEncodeVocab?ra=encode%2Fcv.ra&term=%22A549%22
HepG2	hepatocellular carcinoma	liver	https://genome.ucsc.edu/cgi-bin/hgEncodeVocab?ra=encode%2Fcv.ra&term=%22HepG2%22
MCF-7	mammary gland adenocarcinoma	breast	https://genome.ucsc.edu/cgi-bin/hgEncodeVocab?ra=encode%2Fcv.ra&term=%22MCF%2D7%22
Caco-2	large intestine, adenocarcinoma	colon	https://genome.ucsc.edu/cgi-bin/hgEncodeVocab?ra=encode%2Fcv.ra&term=%22Caco%2D2%22
HCT-116	colorectal carcinoma	colon	https://genome.ucsc.edu/cgi-bin/hgEncodeVocab?ra=encode%2Fcv.ra&term=%22HCT%2D116%22
LNCaP	prostate adenocarcinoma	prostate	https://genome.ucsc.edu/cgi-bin/hgEncodeVocab?ra=encode%2Fcv.ra&term=%22LNCaP%22
Ovcar-3	ovarian adenocarcinoma	ovary	https://genome.ucsc.edu/cgi-bin/hgEncodeVocab?ra=encode%2Fcv.ra&term=%22ovcar%2D3%22
PANC-1	pancreatic carcinoma	pancreas	https://genome.ucsc.edu/cgi-bin/hgEncodeVocab?ra=encode%2Fcv.ra&term=%22PANC%2D1%22
PFSK-1	cerebral brain tumor	brain	https://genome.ucsc.edu/cgi-bin/hgEncodeVocab?ra=encode%2Fcv.ra&term=%22PFSK%2D1%22
T-47D	mammary ductal carcinoma	breast	https://genome.ucsc.edu/cgi-bin/hgEncodeVocab?ra=encode%2Fcv.ra&term=%22T%2D47D%22
U87	glioblastoma, astrocytoma	brain	https://genome.ucsc.edu/cgi-bin/hgEncodeVocab?ra=encode%2Fcv.ra&term=%22U87%22

Details on cell lines are from https://genome.ucsc.edu/cgi-bin/hgTrackUi?hgsid=878021343_bibLdVTxAvl8paf1lnSpeD30jwoh&c=chr19&g=wgEncodeHaibMethyl450

Table S4. Significative *KEAP1* CpG site marked in bold in LUSC non-neoplastic cohort.

Correlation between CpG methylation and protein levels	
Pearson's correlation coefficient (R)	*p value*
0,22	0,03
-0,06	0,57
-0,03	0,76
0,10	0,35
0,00	0,99
0,10	0,36
0,13	0,20
0,32	0,00
0,11	0,29
-0,06	0,59
0,06	0,60
-0,15	0,15
-0,19	0,08
-0,02	0,82
-0,06	0,54
-0,08	0,46

Supplemental Table S5.

Results from correlation analysis between KEAP1 methylation levels and expression of NRF2 and ARE-targets in LUAD (**A, B**).

Results from correlation analysis between KEAP1 methylation levels and expression of NRF2 and ARE-targets in LUSC (**C**).

A_LUAD_All stages_NRF2 and ARE-targets.

		NRF2		***GCLC***		***ABCC2***		***PGD***		***GPX2***		***AKR1C1***		***TXNRD1***		***SRXN1***	
**CpG***	**CpG**	**P value**	**R**	**P value**	**R**	**P value**	**R**	**P value**	**R**	**P value**	**R**	**P value**	**R**	**P value**	**R**	**P value**	**R**
1	cg25801292	0,13	0,07	0,03	-0,10	0,69	0,02	0,00	-0,16	0,05	-0,09	0,08	-0,08	0,25	-0,05	0,01	-0,12
2	cg02428100	0,88	-0,01	0,95	0,00	0,31	0,05	0,99	0,00	0,15	0,07	0,90	0,01	0,38	0,04	0,91	0,01
3	cg26500801	0,13	0,07	0,21	-0,06	0,02	0,11	0,37	-0,04	0,61	0,02	0,58	-0,03	0,45	0,04	0,12	-0,07
4	cg03890664	0,57	0,03	0,90	-0,01	0,96	0,00	0,16	-0,07	0,56	-0,03	0,86	-0,01	0,20	0,06	0,23	-0,06
5	cg15676203	0,88	-0,01	0,62	0,02	0,04	0,10	0,85	-0,01	0,01	0,12	0,56	0,03	0,73	0,02	0,28	-0,05
6	cg06911149	0,03	-0,10	0,62	-0,02	0,01	0,11	0,60	-0,03	0,08	0,08	0,91	0,01	0,20	0,06	0,22	-0,06
7	cg15204119	0,41	0,04	0,33	0,05	0,59	-0,03	0,40	0,04	0,43	-0,04	0,24	-0,06	0,89	0,01	0,78	0,01
8	cg26988016	0,99	0,00	0,86	-0,01	0,57	0,03	0,38	0,04	0,38	-0,04	0,15	-0,07	0,02	0,11	0,15	-0,07
9	cg20226327	0,65	-0,02	0,07	-0,09	0,00	-0,17	0,00	-0,19	0,00	-0,36	0,00	-0,31	0,00	-0,15	0,00	-0,16
10	cg10505024	0,57	-0,03	0,23	-0,06	0,00	-0,17	0,00	-0,16	0,00	-0,28	0,00	-0,28	0,17	-0,06	0,03	-0,10
11	cg07695362	0,42	-0,04	0,92	0,00	0,00	-0,18	0,61	-0,02	0,01	-0,12	0,00	-0,14	0,16	-0,07	0,76	0,01
12	cg00522555	0,89	-0,01	0,12	-0,07	0,17	-0,06	0,00	-0,14	0,34	-0,05	0,24	-0,06	0,00	-0,13	0,41	-0,04
13	cg01018726	0,24	-0,06	0,06	-0,09	0,28	-0,05	0,16	-0,07	0,25	-0,05	0,17	-0,06	0,28	-0,05	0,97	0,00
14	cg22779878	0,02	-0,11	0,05	-0,09	0,01	-0,13	0,00	-0,16	0,00	-0,25	0,00	-0,33	0,26	-0,05	0,01	-0,12
15	cg02337283	0,51	0,03	0,62	0,02	0,18	-0,06	0,13	0,07	0,48	0,03	0,43	-0,04	0,64	-0,02	0,01	0,12
16	cg01586432	0,62	-0,02	0,00	-0,20	0,00	-0,26	0,00	-0,13	0,00	-0,26	0,00	-0,26	0,02	-0,11	0,04	-0,10

**CpG number refers to the position of CpGs indicated in the legend of Figure 8.*

B_LUAD_Non-metastatic_NRF2 and ARE-targets.

		NRF2		***GCLC***		***ABCC2***		***PGD***		***GPX2***		***AKR1C1***		***TXNRD1***		***SRXN1***	
**CpG***	**CpG**	P value	R	P value	R	P value	R	P value	R	P value	R	P value	R	P value	R	P value	R
1	cg25801292	0,18	0,12	0,34	-0,09	0,56	0,05	0,32	-0,09	0,01	-0,22	0,02	-0,21	0,48	-0,06	0,00	-0,31
2	cg02428100	0,84	0,02	0,27	0,10	0,10	0,15	0,40	-0,08	0,72	0,03	0,77	-0,03	0,30	0,09	0,32	0,09
3	cg26500801	0,37	0,08	0,79	-0,02	0,72	0,03	0,05	-0,18	0,32	-0,09	0,17	-0,12	0,87	0,02	0,11	-0,15
4	cg03890664	0,35	0,09	0,38	-0,08	0,73	0,03	0,06	-0,17	0,30	-0,09	0,91	0,01	0,10	0,15	0,58	-0,05
5	cg15676203	0,15	0,13	0,70	0,04	0,81	0,02	0,28	-0,10	0,53	0,06	1,00	0,00	0,12	0,14	0,33	-0,09
6	cg06911149	0,79	-0,02	0,92	0,01	0,09	0,15	0,04	-0,18	1,00	0,00	0,32	-0,09	0,08	0,16	0,51	-0,06
7	cg15204119	0,66	0,04	0,69	0,04	0,17	-0,12	0,81	-0,02	0,00	-0,28	0,01	-0,23	0,84	-0,02	0,65	-0,04
8	cg26988016	0,24	0,11	0,63	0,04	0,07	0,17	0,72	-0,03	0,13	-0,14	0,94	-0,01	0,00	0,31	0,63	-0,04
9	cg20226327	0,71	-0,03	0,99	0,00	0,77	-0,03	0,15	-0,13	0,00	-0,27	0,06	-0,17	0,79	-0,02	0,22	-0,11
10	cg10505024	0,27	-0,10	0,33	-0,09	0,19	-0,12	0,54	-0,06	0,01	-0,22	0,04	-0,18	0,99	0,00	0,56	-0,05
11	cg07695362	0,23	-0,11	0,84	0,02	0,24	-0,11	0,67	-0,04	0,23	-0,11	0,28	-0,10	0,33	-0,09	0,79	-0,02
12	cg00522555	0,01	-0,23	0,53	-0,06	0,44	-0,07	0,44	-0,07	0,48	0,07	0,32	-0,09	0,04	-0,19	0,50	0,06
13	cg01018726	0,11	-0,15	0,25	-0,10	0,04	-0,19	0,95	0,01	0,53	0,06	0,42	-0,07	0,10	-0,15	1,00	0,00
14	cg22779878	0,02	-0,21	0,02	-0,22	0,14	-0,13	0,50	-0,06	0,09	-0,16	0,00	-0,27	0,87	0,02	0,42	-0,07
15	cg02337283	0,64	-0,04	0,55	0,05	0,57	-0,05	0,29	0,10	0,57	-0,05	0,04	-0,19	0,88	0,01	0,26	0,10
16	cg01586432	0,71	-0,03	0,01	-0,22	0,00	-0,26	0,17	-0,12	0,16	-0,13	0,05	-0,17	0,79	-0,02	0,66	-0,04

**CpG number refers to the position of CpGs indicated in the legend of Figure 8.*

C_LUSC_All stages_NRF2 and ARE-targets.

		NRF2		***GCLC***		***ABCC2***		***PGD***		***GPX2***		***AKR1C1***		***TXNRD1***		***SRXN1***	
**CpG***	**CpG**	P value	R	P value	R	P value	R	P value	R	P value	R	P value	R	P value	R	P value	R
1	cg25801292	0,00	-0,24	0,00	-0,18	0,66	0,02	0,00	-0,16	0,00	-0,20	0,00	-0,16	0,04	-0,11	0,29	-0,06
2	cg02428100	0,09	-0,09	0,33	0,05	0,97	0,00	0,38	-0,05	0,29	-0,06	0,35	-0,05	0,93	0,00	0,23	-0,06
3	cg26500801	0,15	-0,08	0,99	0,00	0,86	0,01	0,08	-0,09	0,39	-0,05	0,12	-0,08	0,41	-0,04	0,28	-0,06
4	cg03890664	0,78	0,01	0,08	0,09	0,31	0,05	0,72	0,02	0,37	0,05	0,55	0,03	0,24	0,06	0,99	0,00
5	cg15676203	0,04	-0,11	0,89	-0,01	0,35	0,05	0,38	-0,05	0,40	-0,04	0,41	-0,04	0,68	-0,02	0,93	0,00
6	cg06911149	0,30	-0,05	0,52	0,03	0,27	0,06	0,18	-0,07	0,52	-0,03	0,37	-0,05	0,27	-0,06	0,44	-0,04
7	cg15204119	0,23	-0,06	0,52	-0,03	0,79	0,01	0,07	-0,10	0,99	0,00	0,07	-0,09	0,03	-0,11	0,06	-0,10
8	cg26988016	0,99	0,00	0,13	-0,08	0,91	0,01	0,02	-0,12	0,02	-0,12	0,08	-0,09	0,05	-0,10	0,06	-0,10
9	cg20226327	0,00	-0,22	0,00	-0,20	0,01	-0,13	0,00	-0,25	0,00	-0,26	0,00	-0,22	0,00	-0,18	0,00	-0,18
10	cg10505024	0,00	-0,24	0,00	-0,22	0,14	-0,08	0,00	-0,16	0,00	-0,18	0,00	-0,18	0,01	-0,14	0,03	-0,12
11	cg07695362	0,03	-0,11	0,02	-0,12	0,08	-0,09	0,08	-0,09	0,01	-0,14	0,01	-0,13	0,03	-0,11	0,07	-0,09
12	cg00522555	0,63	0,03	0,65	-0,02	0,12	-0,08	0,40	-0,04	0,92	-0,01	0,60	-0,03	0,51	-0,03	0,15	-0,08
13	cg01018726	0,37	0,05	0,37	-0,05	0,79	-0,01	0,42	0,04	0,69	0,02	0,41	0,04	0,73	-0,02	0,89	-0,01
14	cg22779878	0,00	-0,35	0,00	-0,27	0,86	-0,01	0,00	-0,22	0,00	-0,28	0,00	-0,27	0,00	-0,21	0,01	-0,14
15	cg02337283	0,11	-0,08	0,02	-0,12	0,05	-0,10	0,43	-0,04	0,02	-0,12	0,02	-0,13	0,07	-0,10	0,53	-0,03
16	cg01586432	0,03	-0,11	0,14	-0,08	0,09	0,09	0,53	0,03	0,87	-0,01	0,97	0,00	0,34	0,05	0,07	0,09

Supplemental Table S6. Results from correlation analysis between *KEAP1* methylation levels and expression of NRF2 and ARE-targets in LUAD and LUSC *EGFR/KRAS* subpopulations.

Results from correlation analysis between KEAP1 methylation levels and expression of NRF2 and ARE-targets in EGFR wild type/mutated in LUAD early stages (**A, B**).

Results from correlation analysis between KEAP1 methylation levels and expression of NRF2 and ARE-targets in EGFR wild type/mutated in LUAD all stages (**C, D**).

Results from correlation analysis between KEAP1 methylation levels and expression of NRF2 and ARE-targets in KRAS wild type/mutated in LUAD early stages (**E, F**).

Results from correlation analysis between KEAP1 methylation levels and expression of NRF2 and ARE-targets in KRAS wild type/mutated in LUAD all stages (**G, H**).

Results from correlation analysis between KEAP1 methylation levels and expression of NRF2 and ARE-targets in KRAS wild type/mutated in LUSC all stages (**I, L**).

A) LUAD_EGFR_wild type_early_stages

		NRF2		*GCLC*		*ABCC2*		*PGD*		*GPX2*		*AKR1C1*		*TXNRD1*		*SRXN1*	
**CpG***	**CpG**	P value	R	P value	R	P value	R	P value	R	P value	R	P value	R	P value	R	P value	R
1	cg25801292	0,33	1,00	0,33	-1,00	0,33	-1,00	0,33	-1,00	0,33	-1,00	0,33	-1,00	0,33	-1,00	0,33	-1,00
2	cg02428100	1,00	0,50	1,00	-0,50	1,00	-0,50	1,00	-0,50	1,00	-0,50	1,00	-0,50	1,00	-0,50	1,00	-0,50
3	cg26500801	1,00	0,50	1,00	-0,50	1,00	-0,50	1,00	-0,50	1,00	-0,50	1,00	-0,50	1,00	-0,50	1,00	-0,50
4	cg03890664	1,00	0,50	1,00	-0,50	1,00	-0,50	1,00	-0,50	1,00	-0,50	1,00	-0,50	1,00	-0,50	1,00	-0,50
5	cg15676203	1,00	0,50	1,00	-0,50	1,00	-0,50	1,00	-0,50	1,00	-0,50	1,00	-0,50	1,00	-0,50	1,00	-0,50
6	cg06911149	1,00	0,50	1,00	-0,50	1,00	-0,50	1,00	-0,50	1,00	-0,50	1,00	-0,50	1,00	-0,50	1,00	-0,50
7	cg15204119	1,00	-0,50	1,00	0,50	1,00	0,50	1,00	0,50	1,00	0,50	1,00	0,50	1,00	0,50	1,00	0,50
8	cg26988016	1,00	0,50	1,00	-0,50	1,00	-0,50	1,00	-0,50	1,00	-0,50	1,00	-0,50	1,00	-0,50	1,00	-0,50
9	cg20226327	1,00	0,50	1,00	-0,50	1,00	-0,50	1,00	-0,50	1,00	-0,50	1,00	-0,50	1,00	-0,50	1,00	-0,50
10	cg10505024	1,00	0,50	1,00	-0,50	1,00	-0,50	1,00	-0,50	1,00	-0,50	1,00	-0,50	1,00	-0,50	1,00	-0,50
11	cg07695362	1,00	0,50	1,00	-0,50	1,00	-0,50	1,00	-0,50	1,00	-0,50	1,00	-0,50	1,00	-0,50	1,00	-0,50
12	cg00522555	0,33	-0,87	0,33	0,87	0,33	0,87	0,33	0,87	1,00	-0,50	0,33	0,87	0,33	0,87	0,33	0,87
13	cg01018726	1,00	-0,50	1,00	0,50	1,00	0,50	1,00	0,50	1,00	0,50	1,00	0,50	1,00	0,50	1,00	0,50
14	cg22779878	1,00	-0,50	1,00	0,50	1,00	0,50	1,00	0,50	1,00	0,50	1,00	0,50	1,00	0,50	1,00	0,50
15	cg02337283	1,00	-0,50	1,00	0,50	1,00	0,50	1,00	0,50	1,00	0,50	1,00	0,50	1,00	0,50	1,00	0,50
16	cg01586432	1,00	-0,50	1,00	0,50	1,00	0,50	1,00	0,50	1,00	0,50	1,00	0,50	1,00	0,50	1,00	0,50

**CpG number refers to the position of CpGs indicated in the legend of Figure 8.*

B) LUAD_EGFR_mutated_early_stages

		NRF2		***GCLC***		***ABCC2***		***PGD***		***GPX2***		***AKR1C1***		***TXNRD1***		***SRXN1***	
**CpG***	**CpG**	P value	R	P value	R	P value	R	P value	R	P value	R	P value	R	P value	R	P value	R
1	cg25801292	0,23	0,11	0,39	-0,08	0,53	0,06	0,43	-0,07	0,02	-0,22	0,02	-0,21	0,61	-0,05	0,00	-0,30
2	cg02428100	0,93	0,01	0,26	0,10	0,10	0,15	0,50	-0,06	0,72	0,03	0,76	-0,03	0,27	0,10	0,29	0,10
3	cg26500801	0,37	0,08	0,77	-0,03	0,75	0,03	0,06	-0,17	0,31	-0,09	0,16	-0,13	0,90	0,01	0,10	-0,15
4	cg03890664	0,43	0,07	0,48	-0,07	0,62	0,05	0,08	-0,16	0,40	-0,08	0,83	0,02	0,04	0,18	0,77	-0,03
5	cg15676203	0,21	0,12	0,67	0,04	0,84	0,02	0,35	-0,09	0,50	0,06	0,98	0,00	0,09	0,16	0,38	-0,08
6	cg06911149	0,67	-0,04	0,86	0,02	0,09	0,16	0,06	-0,17	0,95	0,01	0,34	-0,09	0,06	0,17	0,58	-0,05
7	cg15204119	0,60	0,05	0,73	0,03	0,15	-0,13	0,81	-0,02	0,00	-0,29	0,01	-0,24	0,70	-0,04	0,53	-0,06
8	cg26988016	0,28	0,10	0,48	0,07	0,04	0,19	0,95	-0,01	0,18	-0,12	0,85	0,02	0,00	0,33	0,75	-0,03
9	cg20226327	0,61	-0,05	0,88	0,01	0,81	-0,02	0,22	-0,11	0,00	-0,27	0,07	-0,17	0,85	-0,02	0,26	-0,10
10	cg10505024	0,23	-0,11	0,40	-0,08	0,21	-0,12	0,69	-0,04	0,02	-0,22	0,06	-0,18	0,94	0,01	0,61	-0,05
11	cg07695362	0,24	-0,11	0,73	0,03	0,28	-0,10	0,80	-0,02	0,27	-0,10	0,39	-0,08	0,32	-0,09	0,80	-0,02
12	cg00522555	0,02	-0,22	0,43	-0,07	0,39	-0,08	0,29	-0,10	0,58	0,05	0,25	-0,11	0,02	-0,21	0,62	0,05
13	cg01018726	0,13	-0,14	0,20	-0,12	0,03	-0,20	0,87	-0,01	0,60	0,05	0,35	-0,09	0,07	-0,16	0,90	-0,01
14	cg22779878	0,02	-0,21	0,02	-0,22	0,15	-0,13	0,56	-0,05	0,09	-0,16	0,00	-0,26	0,95	0,01	0,39	-0,08
15	cg02337283	0,65	-0,04	0,66	0,04	0,45	-0,07	0,41	0,08	0,49	-0,06	0,02	-0,22	0,92	0,01	0,28	0,10
16	cg01586432	0,84	-0,02	0,01	-0,23	0,00	-0,27	0,15	-0,13	0,13	-0,14	0,06	-0,17	0,59	-0,05	0,50	-0,06

**CpG number refers to the position of CpGs indicated in the legend of Figure 8.*

C) LUAD_EGFR_wild type_all_stages

		NRF2		*GCLC*		*ABCC2*		*PGD*		*GPX2*		*AKR1C1*		*TXNRD1*		*SRXN1*	
**CpG***	**CpG**	P value	R	P value	R	P value	R	P value	R	P value	R	P value	R	P value	R	P value	R
1	cg25801292	0,06	0,25	0,94	-0,01	0,20	-0,17	0,66	-0,06	0,04	-0,27	0,59	-0,07	0,33	0,13	0,22	0,16
2	cg02428100	0,44	-0,11	0,57	-0,08	0,48	-0,09	0,94	-0,01	0,21	0,17	0,93	0,01	0,32	-0,13	0,24	0,16
3	cg26500801	0,08	-0,24	0,45	-0,10	1,00	0,00	0,62	-0,07	0,85	0,03	0,81	0,03	0,07	-0,24	0,88	-0,02
4	cg03890664	0,85	-0,02	0,63	0,06	0,45	-0,10	0,55	0,08	0,58	0,08	0,21	0,17	0,55	0,08	0,22	0,17
5	cg15676203	0,10	-0,22	0,42	-0,11	0,72	-0,05	0,20	-0,17	0,40	0,11	0,44	-0,10	0,28	-0,14	0,97	0,00
6	cg06911149	0,12	-0,21	0,14	-0,20	0,88	-0,02	0,64	-0,06	0,17	0,18	0,62	-0,07	0,50	-0,09	0,08	0,23
7	cg15204119	0,29	0,14	0,53	0,08	0,02	-0,31	0,95	0,01	0,70	0,05	0,32	0,13	0,61	-0,07	0,52	0,09
8	cg26988016	0,07	-0,24	0,08	-0,23	0,45	-0,10	0,90	-0,02	0,26	-0,15	0,39	-0,12	0,48	-0,10	0,37	-0,12
9	cg20226327	0,22	0,16	0,54	-0,08	0,00	-0,39	0,56	-0,08	0,00	-0,39	0,06	-0,25	0,99	0,00	0,63	0,07
10	cg10505024	0,03	0,28	0,39	-0,12	0,00	-0,44	0,52	-0,09	0,05	-0,26	0,02	-0,30	0,91	-0,02	0,15	0,19
11	cg07695362	0,81	0,03	0,72	-0,05	0,31	-0,14	0,10	-0,22	0,05	-0,26	0,03	-0,28	0,76	-0,04	0,53	0,08
12	cg00522555	0,12	-0,21	0,10	0,22	0,95	0,01	0,61	-0,07	0,46	-0,10	0,80	-0,03	0,74	-0,04	0,15	-0,20
13	cg01018726	0,76	0,04	0,73	0,05	0,71	-0,05	0,92	-0,01	0,36	-0,12	0,45	-0,10	0,49	0,09	0,96	-0,01
14	cg22779878	0,85	0,03	0,27	-0,15	0,37	-0,12	0,60	-0,07	0,57	-0,08	0,18	-0,18	0,64	0,06	0,09	0,23
15	cg02337283	0,18	-0,18	0,32	0,13	0,82	0,03	0,40	0,11	0,28	0,15	0,87	0,02	0,82	0,03	0,22	0,17
16	cg01586432	0,42	0,11	0,09	-0,23	0,03	-0,29	0,58	-0,07	0,15	-0,19	0,32	-0,13	0,82	-0,03	0,29	0,14

**CpG number refers to the position of CpGs indicated in the legend of Figure 8.*

D) LUAD_EGFR_mutated_all_stages

		NRF2		***GCLC***		***ABCC2***		***PGD***		***GPX2***		***AKR1C1***		***TXNRD1***		***SRXN1***	
**CpG***	**CpG**	P value	R	P value	R	P value	R	P value	R	P value	R	P value	R	P value	R	P value	R
1	cg25801292	0,31	0,05	0,02	-0,11	0,36	0,05	0,00	-0,18	0,19	-0,07	0,08	-0,09	0,11	-0,08	0,00	-0,16
2	cg02428100	0,76	0,02	0,80	0,01	0,20	0,07	0,98	0,00	0,28	0,05	0,92	0,00	0,21	0,06	0,76	-0,02
3	cg26500801	0,04	0,10	0,23	-0,06	0,02	0,12	0,43	-0,04	0,65	0,02	0,47	-0,04	0,21	0,06	0,11	-0,08
4	cg03890664	0,72	0,02	0,55	-0,03	0,75	0,02	0,07	-0,09	0,42	-0,04	0,41	-0,04	0,31	0,05	0,07	-0,09
5	cg15676203	0,86	0,01	0,49	0,03	0,02	0,12	0,77	0,01	0,02	0,12	0,39	0,04	0,53	0,03	0,26	-0,06
6	cg06911149	0,07	-0,09	0,81	-0,01	0,01	0,13	0,67	-0,02	0,18	0,07	0,81	0,01	0,15	0,07	0,05	-0,10
7	cg15204119	0,66	0,02	0,54	0,03	0,66	0,02	0,38	0,04	0,35	-0,05	0,09	-0,09	0,80	0,01	0,95	0,00
8	cg26988016	0,71	0,02	0,76	0,02	0,38	0,04	0,32	0,05	0,62	-0,03	0,24	-0,06	0,01	0,14	0,23	-0,06
9	cg20226327	0,39	-0,04	0,07	-0,09	0,00	-0,14	0,00	-0,21	0,00	-0,35	0,00	-0,32	0,00	-0,17	0,00	-0,19
10	cg10505024	0,30	-0,05	0,32	-0,05	0,01	-0,14	0,00	-0,17	0,00	-0,28	0,00	-0,28	0,15	-0,07	0,01	-0,14
11	cg07695362	0,29	-0,05	0,86	0,01	0,00	-0,18	0,87	0,01	0,06	-0,09	0,03	-0,11	0,16	-0,07	0,92	0,00
12	cg00522555	0,61	0,03	0,03	-0,11	0,11	-0,08	0,00	-0,16	0,45	-0,04	0,24	-0,06	0,01	-0,14	0,77	-0,01
13	cg01018726	0,22	-0,06	0,04	-0,10	0,26	-0,06	0,11	-0,08	0,35	-0,05	0,20	-0,06	0,19	-0,07	0,99	0,00
14	cg22779878	0,01	-0,13	0,07	-0,09	0,01	-0,13	0,00	-0,17	0,00	-0,27	0,00	-0,35	0,12	-0,08	0,00	-0,17
15	cg02337283	0,25	0,06	0,84	0,01	0,12	-0,08	0,19	0,07	0,69	0,02	0,38	-0,04	0,57	-0,03	0,02	0,12
16	cg01586432	0,49	-0,04	0,00	-0,21	0,00	-0,26	0,00	-0,14	0,00	-0,27	0,00	-0,27	0,01	-0,13	0,01	-0,13

**CpG number refers to the position of CpGs indicated in the legend of Figure 8.*

E) LUAD_KRAS_wild type_early stages

		NRF2		***GCLC***		***ABCC2***		***PGD***		***GPX2***		***AKR1C1***		***TXNRD1***		***SRXN1***	
**CpG***	**CpG**	P value	R	P value	R	P value	R	P value	R	P value	R	P value	R	P value	R	P value	R
1	cg25801292	0,26	0,11	0,09	-0,17	0,49	0,07	0,24	-0,12	0,06	-0,18	0,01	-0,25	0,42	-0,08	0,00	-0,32
2	cg02428100	0,93	-0,01	0,08	0,17	0,07	0,18	0,71	-0,04	0,95	0,01	0,86	0,02	0,13	0,15	0,65	0,05
3	cg26500801	0,22	0,12	0,68	-0,04	0,99	0,00	0,09	-0,17	0,08	-0,17	0,16	-0,14	0,78	0,03	0,04	-0,20
4	cg03890664	0,88	0,01	0,38	-0,09	0,59	0,05	0,10	-0,16	0,10	-0,16	0,87	-0,02	0,17	0,14	0,19	-0,13
5	cg15676203	0,20	0,13	0,57	0,06	0,54	0,06	0,70	-0,04	0,57	0,06	0,87	0,02	0,06	0,19	0,26	-0,11
6	cg06911149	0,98	0,00	0,53	0,06	0,04	0,20	0,12	-0,15	0,83	-0,02	0,83	-0,02	0,02	0,23	0,33	-0,10
7	cg15204119	0,74	-0,03	0,65	0,04	0,48	-0,07	0,75	-0,03	0,00	-0,31	0,01	-0,27	0,83	-0,02	0,42	-0,08
8	cg26988016	0,32	0,10	0,41	0,08	0,06	0,19	0,70	-0,04	0,19	-0,13	0,81	-0,02	0,00	0,33	0,49	-0,07
9	cg20226327	0,99	0,00	0,78	0,03	0,96	0,00	0,15	-0,14	0,01	-0,26	0,25	-0,11	0,86	0,02	0,20	-0,13
10	cg10505024	0,33	-0,10	0,97	0,00	0,49	-0,07	0,62	-0,05	0,07	-0,18	0,44	-0,08	0,46	0,07	0,40	-0,08
11	cg07695362	0,31	-0,10	0,63	0,05	0,44	-0,08	0,89	-0,01	0,59	-0,05	0,96	0,00	0,75	-0,03	0,84	-0,02
12	cg00522555	0,13	-0,15	0,17	-0,14	0,08	-0,17	0,23	-0,12	0,72	0,04	0,22	-0,12	0,02	-0,24	0,44	0,08
13	cg01018726	0,13	-0,15	0,26	-0,11	0,03	-0,21	0,37	-0,09	0,44	0,08	0,45	-0,08	0,09	-0,17	0,88	-0,01
14	cg22779878	0,04	-0,21	0,11	-0,16	0,18	-0,13	0,41	-0,08	0,09	-0,17	0,01	-0,24	0,39	0,09	0,14	-0,15
15	cg02337283	0,80	-0,03	0,63	0,05	0,46	-0,07	0,59	0,05	0,51	-0,07	0,04	-0,21	0,96	0,01	0,60	0,05
16	cg01586432	0,81	-0,02	0,10	-0,16	0,02	-0,23	0,17	-0,13	0,20	-0,13	0,25	-0,11	0,69	0,04	0,47	-0,07

**CpG number refers to the position of CpGs indicated in the legend of Figure 8.*

F) LUAD_KRAS mutated_early stages

		NRF2		*GCLC*		*ABCC2*		*PGD*		*GPX2*		*AKR1C1*		*TXNRD1*		*SRXN1*	
**CpG***	**CpG**	P value	R	P value	R	P value	R	P value	R	P value	R	P value	R	P value	R	P value	R
1	cg25801292	0,61	0,13	0,23	0,30	0,84	-0,05	0,56	0,15	0,01	-0,61	0,95	-0,02	0,78	-0,07	0,55	-0,15
2	cg02428100	0,54	0,16	0,15	-0,35	0,97	-0,01	0,43	-0,20	0,84	0,05	0,11	-0,39	0,23	-0,30	0,25	0,28
3	cg26500801	0,31	-0,25	0,88	-0,04	0,40	0,21	0,41	-0,21	0,33	0,24	0,51	-0,16	0,43	-0,20	0,54	0,16
4	cg03890664	0,36	0,23	0,23	-0,30	0,50	-0,17	0,49	-0,17	1,00	0,00	0,61	-0,13	0,90	-0,03	0,31	0,25
5	cg15676203	0,86	0,04	0,67	-0,11	0,50	-0,17	0,15	-0,35	0,74	0,09	0,57	-0,14	0,23	-0,30	0,63	0,12
6	cg06911149	0,43	-0,20	0,11	-0,39	0,75	-0,08	0,14	-0,36	0,60	0,13	0,02	-0,55	0,08	-0,42	0,59	0,14
7	cg15204119	0,25	0,29	0,59	-0,14	0,04	-0,49	0,87	-0,04	0,26	-0,28	0,31	-0,25	0,30	-0,26	0,90	-0,03
8	cg26988016	0,37	0,22	0,29	-0,26	0,93	0,02	0,95	-0,02	0,56	-0,15	0,91	0,03	0,86	0,05	0,56	0,15
9	cg20226327	0,40	-0,21	0,17	-0,34	0,37	-0,23	0,66	-0,11	0,12	-0,38	0,02	-0,56	0,14	-0,36	0,86	-0,05
10	cg10505024	0,96	0,01	0,01	-0,58	0,20	-0,32	0,64	-0,12	0,21	-0,31	0,00	-0,67	0,20	-0,31	0,47	0,18
11	cg07695362	0,82	-0,06	0,34	-0,24	0,12	-0,38	0,48	-0,18	0,11	-0,39	0,03	-0,51	0,05	-0,47	0,97	0,01
12	cg00522555	0,00	-0,66	0,07	0,44	0,03	0,51	0,44	0,20	0,20	0,32	0,61	0,13	0,38	0,22	0,93	0,02
13	cg01018726	0,44	0,20	0,95	0,02	0,37	-0,22	0,29	0,26	0,78	0,07	0,66	0,11	0,69	0,10	0,65	0,11
14	cg22779878	0,52	-0,16	0,01	-0,57	0,84	-0,05	0,81	-0,06	0,70	0,10	0,10	-0,40	0,20	-0,32	0,27	0,28
15	cg02337283	0,39	-0,22	0,94	0,02	0,49	0,18	0,08	0,42	0,76	-0,08	0,72	-0,09	0,76	0,08	0,07	0,43
16	cg01586432	0,94	0,02	0,01	-0,58	0,19	-0,32	0,45	-0,19	0,91	-0,03	0,04	-0,50	0,09	-0,41	0,58	0,14

**CpG number refers to the position of CpGs indicated in the legend of Figure 8.*

G) LUAD_KRAS wild type_all stages

		NRF2		*GCLC*		*ABCC2*		*PGD*		*GPX2*		*AKR1C1*		*TXNRD1*		*SRXN1*	
**CpG***	**CpG**	P value	R	P value	R	P value	R	P value	R	P value	R	P value	R	P value	R	P value	R
1	cg25801292	0,03	-0,11	0,09	0,09	0,00	1,00	0,00	0,17	0,11	-0,08	0,01	-0,13	0,88	0,01	0,01	0,14
2	cg02428100	0,89	0,01	0,96	0,00	0,03	0,11	0,81	0,01	0,00	-0,33	0,00	-0,23	0,54	-0,03	1,00	0,00
3	cg26500801	0,01	-0,12	0,18	-0,07	0,03	0,11	0,27	-0,06	0,00	-0,23	0,01	-0,13	0,00	-0,23	0,22	-0,06
4	cg03890664	0,22	-0,06	0,77	-0,02	0,02	0,12	0,47	0,04	0,00	-0,30	0,00	-0,34	0,70	-0,02	0,83	0,01
5	cg15676203	0,03	-0,11	0,27	-0,06	0,03	0,11	0,97	0,00	0,00	-0,39	0,00	-0,36	0,43	-0,04	0,68	-0,02
6	cg06911149	0,01	-0,13	0,17	-0,07	0,00	0,17	0,66	0,02	0,00	-0,40	0,00	-0,35	0,32	-0,05	0,96	0,00
7	cg15204119	0,03	0,11	0,48	-0,04	0,63	0,02	0,58	0,03	0,02	-0,12	0,00	-0,17	0,09	0,09	0,80	0,01
8	cg26988016	0,04	-0,10	0,14	0,07	0,00	0,16	0,01	0,14	0,00	-0,26	0,00	-0,33	0,95	0,00	0,02	0,11
9	cg20226327	0,76	0,02	0,00	0,42	0,00	0,19	0,00	0,49	0,32	0,05	0,72	-0,02	0,00	0,29	0,00	0,78
10	cg10505024	0,68	0,02	0,00	0,48	0,01	0,14	0,00	0,65	0,21	0,06	0,07	-0,09	0,00	0,37	0,00	1,00
11	cg07695362	0,00	0,18	0,00	0,26	0,88	0,01	0,00	0,31	0,15	0,07	0,01	-0,14	0,00	1,00	0,00	0,37
12	cg00522555	0,00	0,23	0,25	0,06	0,01	-0,13	0,06	-0,10	0,00	0,45	0,00	1,00	0,01	-0,14	0,07	-0,09
13	cg01018726	0,00	0,20	0,04	0,10	0,11	-0,08	0,56	0,03	0,00	1,00	0,00	0,45	0,15	0,07	0,21	0,06
14	cg22779878	0,06	0,10	0,00	0,50	0,00	0,17	0,00	1,00	0,56	0,03	0,06	-0,10	0,00	0,31	0,00	0,65
15	cg02337283	0,00	1,00	0,01	0,13	0,03	-0,11	0,06	0,10	0,00	0,20	0,00	0,23	0,00	0,18	0,68	0,02
16	cg01586432	0,01	0,13	0,00	1,00	0,09	0,09	0,00	0,50	0,04	0,10	0,25	0,06	0,00	0,26	0,00	0,48

**CpG number refers to the position of CpGs indicated in the legend of Figure 8.*

H) LUAD_KRAS mutated_all stages

		NRF2		*GCLC*		*ABCC2*		*PGD*		*GPX2*		*AKR1C1*		*TXNRD1*		*SRXN1*	
**CpG***	**CpG**	P value	R	P value	R	P value	R	P value	R	P value	R	P value	R	P value	R	P value	R
1	cg25801292	0,57	0,07	0,82	0,03	0,53	-0,08	1,00	0,00	0,05	-0,25	0,73	-0,04	0,83	0,03	0,91	-0,01
2	cg02428100	0,18	-0,17	0,75	-0,04	0,64	0,06	0,64	0,06	0,25	0,15	0,96	-0,01	0,72	-0,05	0,08	0,23
3	cg26500801	0,10	-0,21	0,85	-0,02	0,70	0,05	0,99	0,00	0,77	0,04	0,97	-0,01	0,43	-0,10	0,22	0,16
4	cg03890664	0,23	0,16	0,68	0,05	0,30	-0,13	0,43	0,10	0,89	-0,02	0,54	0,08	0,42	0,10	0,08	0,22
5	cg15676203	0,57	-0,07	0,23	-0,16	0,71	-0,05	0,33	-0,13	0,48	0,09	0,81	-0,03	0,46	-0,10	0,31	0,13
6	cg06911149	0,02	-0,30	0,07	-0,23	0,32	-0,13	0,21	-0,16	0,75	0,04	0,02	-0,30	0,32	-0,13	0,21	0,16
7	cg15204119	0,61	0,07	0,89	-0,02	0,07	-0,23	0,78	0,04	0,79	0,03	0,46	0,10	0,49	-0,09	0,56	0,07
8	cg26988016	0,88	-0,02	0,37	-0,12	0,50	-0,09	0,18	0,17	0,20	-0,16	0,71	-0,05	0,88	-0,02	0,74	0,04
9	cg20226327	0,86	0,02	0,30	-0,13	0,18	-0,17	0,90	-0,02	0,09	-0,22	0,02	-0,30	0,13	-0,20	0,68	0,05
10	cg10505024	0,88	0,02	0,35	-0,12	0,19	-0,17	0,99	0,00	0,28	-0,14	0,01	-0,32	0,39	-0,11	0,38	0,11
11	cg07695362	0,73	-0,04	0,15	-0,18	0,01	-0,35	0,12	-0,20	0,02	-0,29	0,01	-0,35	0,03	-0,27	0,92	-0,01
12	cg00522555	0,05	-0,25	0,35	0,12	0,49	0,09	0,48	-0,09	0,98	0,00	0,41	-0,11	0,90	0,02	0,45	-0,10
13	cg01018726	0,61	0,07	0,97	0,01	0,96	-0,01	0,63	0,06	0,73	0,05	0,80	0,03	0,40	0,11	0,67	0,05
14	cg22779878	0,35	-0,12	0,18	-0,17	0,41	-0,11	0,84	0,03	0,79	-0,03	0,18	-0,17	0,37	-0,12	0,29	0,14
15	cg02337283	0,28	-0,14	0,69	-0,05	0,79	0,03	0,07	0,23	0,32	0,13	0,39	0,11	0,19	0,17	0,33	0,12
16	cg01586432	0,63	0,06	0,02	-0,30	0,01	-0,31	0,31	-0,13	0,26	-0,14	0,10	-0,21	0,05	-0,25	0,57	0,07

**CpG number refers to the position of CpGs indicated in the legend of Figure 8.*

I) LUSC_KRAS_wild type_all stages

		NRF2		*GCLC*		*ABCC2*		*PGD*		*GPX2*		*AKR1C1*		*TXNRD1*		*SRXN1*	
**CpG***	**CpG**	P value	R	P value	R	P value	R	P value	R	P value	R	P value	R	P value	R	P value	R
1	cg25801292	0,00	-0,24	0,00	-0,18	0,59	0,03	0,00	-0,16	0,00	-0,20	0,00	-0,16	0,04	-0,11	0,31	-0,05
2	cg02428100	0,10	-0,09	0,54	0,03	0,74	-0,02	0,28	-0,06	0,16	-0,07	0,25	-0,06	0,69	-0,02	0,18	-0,07
3	cg26500801	0,11	-0,09	0,82	-0,01	0,88	-0,01	0,05	-0,10	0,24	-0,06	0,08	-0,09	0,36	-0,05	0,29	-0,06
4	cg03890664	0,91	-0,01	0,17	0,07	0,46	0,04	0,96	0,00	0,60	0,03	0,78	0,02	0,37	0,05	0,79	-0,01
5	cg15676203	0,02	-0,12	0,50	-0,04	0,55	0,03	0,20	-0,07	0,21	-0,07	0,25	-0,06	0,45	-0,04	0,77	-0,02
6	cg06911149	0,26	-0,06	0,72	0,02	0,46	0,04	0,09	-0,09	0,36	-0,05	0,31	-0,06	0,20	-0,07	0,41	-0,04
7	cg15204119	0,11	-0,09	0,33	-0,05	0,96	0,00	0,02	-0,12	0,66	-0,02	0,03	-0,11	0,01	-0,13	0,04	-0,11
8	cg26988016	0,84	-0,01	0,12	-0,08	0,94	0,00	0,02	-0,12	0,02	-0,13	0,08	-0,09	0,06	-0,10	0,07	-0,10
9	cg20226327	0,00	-0,23	0,00	-0,20	0,02	-0,12	0,00	-0,25	0,00	-0,26	0,00	-0,22	0,00	-0,18	0,00	-0,18
10	cg10505024	0,00	-0,23	0,00	-0,22	0,19	-0,07	0,00	-0,16	0,00	-0,18	0,00	-0,18	0,01	-0,14	0,03	-0,11
11	cg07695362	0,04	-0,11	0,02	-0,12	0,14	-0,08	0,10	-0,09	0,01	-0,14	0,02	-0,13	0,04	-0,11	0,09	-0,09
12	cg00522555	0,40	0,04	0,91	-0,01	0,14	-0,08	0,57	-0,03	0,78	0,01	0,81	-0,01	0,68	-0,02	0,23	-0,06
13	cg01018726	0,21	0,07	0,65	-0,02	0,93	0,00	0,21	0,07	0,37	0,05	0,23	0,06	0,97	0,00	0,88	0,01
14	cg22779878	0,00	-0,34	0,00	-0,26	0,93	0,00	0,00	-0,21	0,00	-0,27	0,00	-0,26	0,00	-0,20	0,01	-0,13
15	cg02337283	0,08	-0,10	0,03	-0,11	0,08	-0,09	0,50	-0,04	0,03	-0,12	0,01	-0,13	0,08	-0,09	0,52	-0,03
16	cg01586432	0,09	-0,09	0,19	-0,07	0,08	0,09	0,39	0,05	0,95	0,00	0,87	0,01	0,32	0,05	0,05	0,10

**CpG number refers to the position of CpGs indicated in the legend of Figure 8.*

L) LUSC_KRAS mutated_all stages

		NRF2		*GCLC*		*ABCC2*		*PGD*		*GPX2*		*AKR1C1*		*TXNRD1*		*SRXN1*	
**CpG***	**CpG**	P value	R	P value	R	P value	R	P value	R	P value	R	P value	R	P value	R	P value	R
1	cg25801292	0,70	-0,11	0,43	-0,22	0,57	-0,16	0,66	0,13	0,98	0,01	0,70	-0,11	0,68	0,12	0,83	-0,06
2	cg02428100	0,53	-0,18	0,18	0,37	0,08	0,47	0,49	0,19	0,55	0,17	0,40	0,24	0,35	0,26	0,90	0,04
3	cg26500801	0,86	0,05	0,59	0,15	0,10	0,44	0,44	0,21	0,70	0,11	0,62	0,14	0,99	0,00	0,60	-0,15
4	cg03890664	0,22	0,34	0,05	0,52	0,22	0,34	0,09	0,45	0,26	0,31	0,08	0,47	0,17	0,38	0,31	0,28
5	cg15676203	0,55	0,17	0,03	0,56	0,05	0,52	0,13	0,41	0,21	0,34	0,15	0,39	0,23	0,33	0,61	0,14
6	cg06911149	0,96	0,01	0,28	0,30	0,11	0,44	0,29	0,29	0,52	0,18	0,59	0,15	0,84	0,06	0,98	-0,01
7	cg15204119	0,19	0,36	0,09	0,46	0,06	0,49	0,02	0,62	0,12	0,42	0,10	0,45	0,34	0,26	0,35	0,26
8	cg26988016	0,28	0,30	0,75	0,09	0,70	0,11	0,73	0,10	0,94	-0,02	0,98	0,01	0,71	-0,10	0,82	-0,06
9	cg20226327	0,43	-0,22	0,14	-0,40	0,06	-0,50	0,18	-0,36	0,09	-0,45	0,08	-0,47	0,19	-0,36	0,25	-0,32
10	cg10505024	0,17	-0,38	0,24	-0,33	0,31	-0,28	0,33	-0,27	0,52	-0,18	0,33	-0,27	0,97	-0,01	0,92	0,03
11	cg07695362	0,63	-0,14	0,96	-0,01	0,09	-0,45	0,62	-0,14	0,74	-0,09	0,80	-0,07	0,97	0,01	0,74	-0,09
12	cg00522555	0,15	-0,39	0,09	-0,45	0,45	-0,21	0,10	-0,44	0,16	-0,38	0,13	-0,41	0,21	-0,34	0,30	-0,28
13	cg01018726	0,22	-0,34	0,05	-0,52	0,31	-0,28	0,08	-0,46	0,26	-0,31	0,09	-0,46	0,22	-0,34	0,30	-0,28
14	cg22779878	0,01	-0,69	0,07	-0,48	0,65	-0,13	0,01	-0,63	0,04	-0,54	0,05	-0,53	0,20	-0,35	0,15	-0,39
15	cg02337283	0,45	0,21	0,59	-0,15	0,35	-0,26	0,71	-0,10	0,99	0,00	0,84	0,06	0,91	0,03	0,74	0,09
16	cg01586432	0,02	-0,59	0,19	-0,36	0,83	-0,06	0,18	-0,37	0,60	-0,15	0,36	-0,25	0,72	-0,10	0,78	-0,08

**CpG number refers to the position of CpGs indicated in the legend of Figure 8.*
